# Graph-Based Modeling of Behavioral Transitions for Digital Health Applications: Trajectory Analysis Framework

**DOI:** 10.2196/86629

**Published:** 2026-09-04

**Authors:** Sezin Kircali Ata, Weizhuang Zhou, Jesisca Tandi, Wei Qing Lee, Yu En Chan, Feri Guretno, Anitha Veeramani, Wei Liu, Jeremy Tan, Felix B Wijaya, Nicole Lim, Pavitra Krishnaswamy, Mojisola Erdt

**Affiliations:** 1 Agency for Science, Technology and Research (A*STAR), Institute for Infocomm Research (I2R), 1 Fusionopolis Way, #21-01 Connexis South Tower, Singapore, 138632, Singapore; 2 Health Promotion Board, Singapore, Singapore

**Keywords:** trajectory analysis, digital health, behavioral informatics, digital health analytics, user behavior, behavior change

## Abstract

**Background:**

Digital health applications generate rich behavioral data; yet, how users transition between behavioral states remains poorly understood. Existing approaches show limited capacity to capture and represent the evolving and nuanced nature of real-world behavioral dynamics, which are essential for informing personalized behavior change interventions.

**Objective:**

This study aimed to understand how users’ behaviors evolve within digital health applications by developing a data-driven approach that captures transition dynamics across behavioral features. We aimed to validate modeled transitions against observed user data, analyze transition pathways, and derive interpretable insights.

**Methods:**

We analyzed 32 weeks of data from 36,574 users from a population health program run by the Health Promotion Board (HPB) in Singapore. We developed a graph-based behavioral trajectory model (GraphBeTraM*)* that models behavioral transitions as shortest paths through user similarity graphs. By aggregating paths over time, the model captures users’ progression toward target behavioral states and quantifies the direction, magnitude, and timing of change. We validated modeled transitions against real-world data by comparing (1) transition matrices and (2) feature-level pathways, using Spearman correlation, Fisher z-transformed means, and cosine distance. We focused on high-variance features and used heatmaps to visualize patterns of change. Finally, characterized transition pathways between behavioral states and distinguished generalizable and context-specific features.

**Results:**

We observed strong alignment between observed and modeled transition matrices with strong correlation (Spearman ρ=0.82; *P*<.001) and low cosine distance (0.05), suggesting that real-world behavioral transitions can be effectively represented through shortest paths with GraphBeTraM. Feature-level pathway validation showed consistent patterns of change across both personalized and real-world pathways. Physical activity features like weekly moderate to vigorous physical activity (MVPA) emerged as stable, generalizable signals of change at the population level, with strong mean correlation (ρ__personalized_=0.97; ρ__real-world_=0.79) and low mean cosine distance (cosine__personalized_=0.24; cosine__real-world_=0.41) across both personalized and real-world transitions. In contrast, features related to personal preferences, including time-related activity patterns (eg, proportion of weekday to weekend MVPA), purchase preferences for healthy foods and drinks, and engagement indicators (eg, last contact with the program or app), exhibited context-specific relevance and reflected change at an individual level. An in-depth transition analysis from an active state with prolonged sedentary periods to an active state with healthy eating habits revealed interpretable patterns in onset, magnitude, and rate of change. Specifically, features such as preferences for healthy food and drink purchases emerged later in the trajectory but then showed sharper and faster transitions once these behaviors began to shift. These insights highlight how GraphBeTraM can guide nudging strategies in alignment with natural behavior dynamics, including both stable and later-onset patterns.

**Conclusions:**

GraphBeTraM provides an interpretable framework for modeling behavioral transitions in digital health applications and captures key dynamics that support personalized intervention design.

## Introduction

Digital health applications play an important role in promoting behavior change across many health domains, including chronic disease management [1], mental health [2], and health education and health promotion [3]. A primary focus of many of these applications is to encourage physical activity, a health-related behavior associated with the prevention and management of noncommunicable diseases, as well as the improvement of mental health [4]. Physical inactivity is a global health concern [5], and digital health interventions that track and influence health behaviors have become very important.

Characterizing and quantifying behavior change is necessary for designing impactful intervention strategies. Methods for assessing behavior change usually comprise self-report measures through surveys and questionnaires [6,7] or real-time monitoring with wearable sensors, activity trackers, smartphone usage patterns, and other digital logs [8-10]. Recent studies have shown the efficacy of objective monitoring in enhancing digital health interventions [11-14]. These approaches provide insights into behavioral patterns but often do not provide in-depth explanations of how user behaviors evolve over time. Existing modeling approaches interpret, structure, and simulate behavior change across varying levels of abstraction and complexity. First, traditional theoretical models, such as the transtheoretical model or the stages of change model [15], and the theory of planned behavior [16] aim to describe behavior change using constructs such as intention and perceived control; however, they lack the ability to capture the dynamics of behavior change in digital applications [17]. Second, behavior-monitoring and habit-detection models use sensor data from wearables or mobile devices to observe behavioral patterns [11,18-22], but these often focus on short-term changes and are not readily generalizable for trajectory analysis. Third, AI-driven adaptive models, such as just-in-time adaptive interventions, use real-time data to deliver interventions tailored to a user’s current context [23-25]. However, they often rely on predefined behavior goals. Fourth, data-driven behavioral pattern discovery models [26-37] use large-scale data to identify patterns of change, particularly in longitudinal health studies. In contrast to behavior monitoring and habit detection, which focus on high-resolution, momentary tracking, these approaches offer a broader and contextually rich understanding of long-term behavioral dynamics. They are empirical in nature and do not rely on predefined goals or theoretical constructs; however, they often require parametric assumptions and do not explicitly model the dynamics of behavioral transitions that are necessary to capture the nuanced and evolving nature of real-world behavioral trajectories. We position the proposed framework within this category of data-driven approaches while addressing the outlined limitations.

This study aims to investigate how users’ behaviors evolve over time in real-world digital health applications, with the goal to understand and characterize behavioral trajectories to inform personalized behavior change strategies. We introduce a graph-based behavioral trajectory model (GraphBeTraM), a graph-based framework that models behavioral trajectories as shortest paths through local neighborhoods in a user similarity graph, where clusters or states represent groups of users with similar behavior and central cluster or state nodes serve as anchors for modeling users’ potential transition pathways. To assess real-world relevance, we aimed to validate these modeled transitions against observed user data and analyze feature transition pathways to distinguish generalizable features that are consistent signals of change across users from those context-specific features that vary across individuals or transitions. Finally, we aim to characterize user pathways toward representative behavioral states, capturing the direction, magnitude, and timing of change, in order to gain insights that could inform the design of targeted and personalized digital health interventions.

## Methods

### Study Design and Dataset

Our study focused on a secondary analysis of data from users aged 21 years and older on the Healthy 365 (Health Promotion Board [HPB]) mobile app with complete data at each time point from baseline on October 30, 2023, till the timeframe of July 1, 2024. Healthy 365 is a free health and wellness tracking app developed by the HPB in Singapore to encourage users to adopt a healthier lifestyle through digital offerings, gamification, and rewards [38]. This resulted in a total of N=36,574 users for our study. For each week during this 32-week timeframe, users were identified as being in one of the behavioral states listed in Table 1. These 7 behavioral states were derived from a segmentation model developed prior to this study as part of operational analysis. The segmentation model was based on 16 behavioral features (see Table 2) that generated these 7 robust and distinct behavioral states, effectively capturing variations in physical activity and engagement levels.

**Table 1.** Description of behavioral states.

Behavioral state	Description
MVPA^a^-focused active	An active state with elevated levels of MVPA
Step-focused active	An active state with elevated levels of steps
Predominantly sedentary	An inactive state with elevated levels of sedentary duration
Highly active	A very active state with very high levels of MVPA
Sedentary office active	A somewhat active state often with notable periods of repeated sedentary duration
Active and healthy eating	An active state with healthy eating habits
Class-based active	A somewhat active state predominantly attending guided exercise classes

a MVPA: moderate to vigorous physical activity.

**Table 2.** Description of behavioral features in the dataset.

Feature	Description
Tracker usage	Average total days with at least 1000 steps in a week over 4 weeks
Ratio sedentary to steps	Proportion of steps to sedentary duration
Weekly MVPA^a^	Average weekly MVPA duration over 4 weeks
Ratio steps to MVPA	Proportion of steps to MVPA
Last contact	Number of days since last contact with program, app, or tracker
Sedentary routine	Average sedentary duration between 8 AM and 11 PM in the past 4 weeks
MVPA on weekends	MVPA during weekends
Ratio weekday to weekend	Proportion of MVPA weekday to weekend
Healthy food and drink purchased	Total number of healthy food and drinks items purchased in last 4 weeks
Types of exercise classes attended	Number of leisure time physical activity types attended in the month
Tracker syncing	Average of total days user synced tracker data in a week over 4 weeks
Healthy groceries purchased	Total number of healthy grocery items purchased in last 4 weeks
MVPA on weekdays	MVPA during weekdays
MVPA trend	Trend of weekly MVPA duration over 4 weeks
Steps tracked	Average daily steps (with >=1000 steps) over 4 weeks
Ratio VPA to MVPA	Proportion of vigorous physical activity (VPA) to MVPA

a MVPA: moderate to vigorous physical activity.

In this study, we developed the GraphBeTraM framework to model user transitions between behavioral states. We validated this model through transition matrices and feature transition pathways, using both personalized shortest path analysis and real-world user transition pathways. Finally, we analyzed the resulting modeled transitions between behavioral states for insights toward behavior change strategies.

### GraphBeTraM Framework

#### Framework Overview

**Figure 1.**
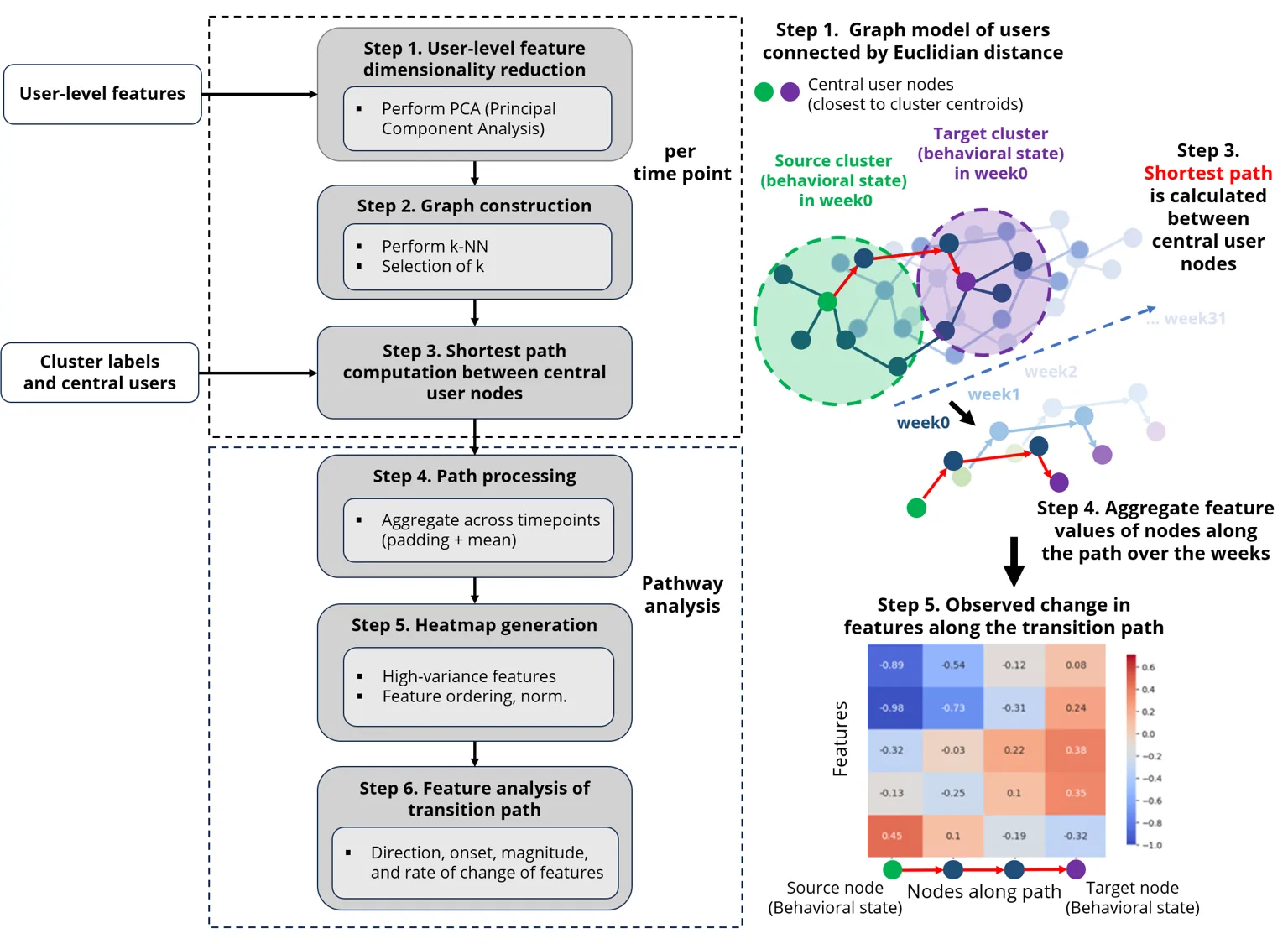
Block diagram of graph-based behavioral trajectory model (GraphBeTraM) showing the main steps (left) and (right) illustration of block diagram steps.

GraphBeTraM is a graph-based framework designed to identify and characterize behavioral change by modeling transitions between clusters as shortest paths through neighboring behavior states in a user similarity graph. By aggregating these paths over successive weeks, the approach captures the direction, magnitude, and timing of feature-level changes, providing an interpretable view of user evolution over time. As illustrated in Figure 1, the framework takes user-level features, cluster labels (ie, behavioral states), and central users (ie, the user closest to the cluster centroid) as input, performs user-level feature dimensionality reduction (step 1), and constructs user similarity graphs in graph construction (step 2), then shortest path computation between central user nodes to model targeted transitions between clusters (step 3). This procedure (steps 1, 2, and 3) is repeated for each time point (week 0-week 31), and the resulting paths are aggregated across 32 weeks in path processing (step 4). Heatmap generation (step 5) visualizes transition pathways from source to target nodes, with columns representing the hypothetical steps required to reach the target node. These representative steps or nodes do not indicate time points, nor do they reflect any intermediary states along the path. Finally, in the feature analysis of the transition path (step 6), the features along the transition paths are analyzed to generate insights. All steps up to and including shortest path computation (steps 1, 2, and 3) are applied at each time point (ie, weekly), while feature aggregation and analysis of the evolution of user features along these paths are performed afterward (steps 4, 5, and 6) across all time points.

#### User-Level Feature Dimensionality Reduction

At each time point *t* ∈ {1,2,..., *T*}, we represent user-level data as a feature matrix *X^t^* ∈ *R^n^*_*^t^*_^×^*^d^*, where *n_t_* is the number of users at *t* and *d* is the number of behavioral features. To ensure comparability and reduce noise in high-dimensional behavioral data, we apply principal component analysis (PCA), obtaining a lower-dimensional embedding:


$$Z^{t}\text{=}\text{PCA}\left(X^{t}\right),\text{where}Z^{t}\in R^{n_{t}\times d^{`}}$$

such that the top *d′* components preserve at least 95% of the variance in *X*^*t*^. Here, the PCA-reduced feature matrix *Z*^*t*^ contains the lower-dimensional representations of users at time *t*, and each user vector *z^t^*_i_ ∈ *Z*^*t*^ represents the behavioral state vector for user *i* at time point *t* in the reduced space. This step serves two conceptual purposes: (1) it captures the most informative behavioral variation while reducing the impact of irrelevant or noisy information, and (2) it enables more reliable user comparisons and clustering by providing a lower-dimensional representation of behavioral states.

#### Graph Construction

A k-nearest neighbor (k-NN) graph *G*^*t*^=(*V*^*t*^,*E*^*t*^) is constructed whereby each node *v^t^*_i_ ∈ *V*^*t*^ corresponds to a user *i* at time point *t*. The node features are given by the corresponding behavioral state vector *z^t^*_i_ ∈ *Z*^*t*^. Edges *E*^*t*^ are formed by connecting each node to its k-NNs based on Euclidean distance in the feature space:


$$e_{ij}^{t}\in E^{t}⟺\|z_{i}^{t}\text{-}z_{j}^{t}{\|}_{2}\text{is among the k smallest for}\;z_{i}^{t}$$

The value of k was empirically selected for each week to ensure stable neighborhood formation. We apply topological clustering using the greedy modularity optimization method implemented in *NetworkX* Python (version 3.3; Python Software Foundation) package to each week’s user similarity graph. We observe that as k increases, the number of detected topological clusters stabilizes. We then choose the smallest k that results in a stable number of neighborhoods for that week, which was approximately k*=*15.

#### Shortest Path Computation Between Central User Nodes

To model likely behavioral transitions, we compute the shortest paths between all pairs of cluster center nodes in the user similarity graph *G*_*t*_ constructed in step B for each time point t. We define shortest paths as plausible minimal sequences of gradual changes a user might take to move from one behavioral state to another, assuming users change step-by-step through local shifts in behavior rather than making abrupt jumps. These shortest paths thus represent potential behavioral transition pathways and approximate how users move between distinct clusters over time. We note that shortest paths do not directly represent observed real-world transitions. We therefore model behavioral change as transitions along the shortest and most behaviorally similar paths. Given cluster assignments *C*_*t*_=, where *K* is the number of clusters and *t* denotes the time point, the shortest path distance is computed between 2 cluster centers *c^t^*_i_ ∈ *C^t^*_i_ and *c^t^*_*j*_ ∈ *C^t^_j_*, which represent the center nodes of clusters *i* and *j* at time point *t*. These nodes correspond to PCA-transformed feature vectors ${z^{t}}_{c_{i}^{t}},{z^{t}}_{c_{j}^{t}}\in Z^{t}$. The distance between them is computed as the sum of edge weights (Euclidean distances in PCA space) along the minimum-cost path:


$$d_{ij}^{t}\text{=}\underset{\text{path}}{min}\sum_{\left(v_{r}^{t},v_{s}^{t}\right)\in \text{path}}{\left\|z_{r}^{t}\text{-}z_{s}^{t}\right\|}_{2}$$

Here *v^t^_r_* and *v*^*t*^_*s*_ are users (nodes) in the path from *c*^*t*^_*i*_ to *c*^*t*^_*j*_. Their corresponding PCA-transformed feature vectors *z*^*t*^_*r*_ , *z^t^*_s_ ∈ *Z*^*t*^ are of users v^t^_r_ and *v*^*t*^_*s*_.

#### Path Processing

Feature vectors of users along each shortest path were collected at each time point. Then the feature values were aggregated by calculating the mean. As path lengths may vary over time, we applied padding to standardize them to the average path length.

### General Pathway Analysis: Population-Level Behavioral Patterns

We conducted an aggregate analysis of transition pathways across the user population to understand general behavioral patterns across shortest paths between cluster center nodes. This analysis gives a high-level understanding of common behavioral trajectories and provides insights into features that vary the most during user transitions.

#### Heatmap Generation

The aggregated feature vectors from all center-to-center paths were first compiled into a matrix, normalized to the interval (−1 to 1), and then visualized as a heatmap. Hierarchical clustering was used to order the features in the heatmap based on the similarity in their transition patterns across the path.

#### Feature Analysis of Transition Path

We characterized behavioral dynamics by calculating the direction, magnitude, and relative timing of change across features along the path. For each feature, the onset index was determined as the earliest step in the path when the feature value crossed zero, which indicates a potential point of change in behavioral direction. The rate of change (RoC) was calculated as the average per-step difference between the final and initial feature values across the path, defined as:


$$\text{RoC}\text{=}\frac{z_{c_{j}^{t}}^{t}\text{-}z_{c_{i}^{t}}^{t}}{m\text{-}1}$$

where $z_{c_{i}^{t}}^{t}$ and $z_{c_{j}^{t}}^{t}$ are normalized features, *c*^*t*^_*i*_and *c*^*t*^_*j*_ are center nodes of the source clusteri*i* and target cluster *j* at time *t* of the trajectory, respectively, and *m* is the number of nodes or steps along the path. The RoC is categorized as very slow, slow, moderate, fast, or very fast, based on equal-interval bins with thresholds relative to the maximum RoC. When feature values are normalized within the range (−1 to 1), the maximum RoC is calculated as 2/(*m*-1), where *m* is the number of steps along the path. These bin thresholds are then uniformly spaced between 0 and this maximum rate, allowing each feature’s RoC to be consistently and comparatively classified. The magnitude of change was computed as the absolute difference between the final and initial values, $\text{|}z_{c_{j}^{t}}^{t}\text{-}z_{c_{i}^{t}}^{t}\text{|}$, capturing the extent of variation in the feature irrespective of direction. Similar to the approach above for RoC, the magnitude of change is categorized from very small to very large.

### Personalized Pathway Analysis: User-Level Behavioral Patterns

**Figure 2.**
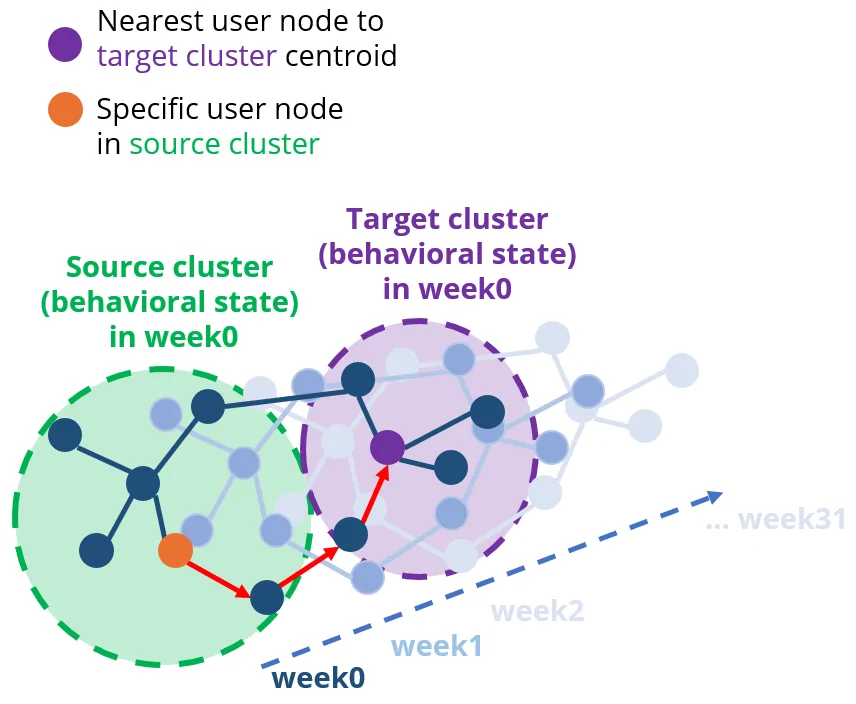
Personalized user-to-center pathway showing the shortest path between a specific user node and the target cluster’s central node. The highlighted red path indicates the trajectory from the source user node (green-labeled cluster) to the target cluster’s central node (purple-labeled cluster) at time point week 0.

GraphBeTraM can also be adapted to personalized transition pathway analysis by modifying the shortest path computation (3) as shown in Figure 2. Specifically, instead of computing shortest paths between all pairs of cluster centers at time point *t*, the source node is redefined as a specific user node *v*^*t*^_*u*_ ∈ *V^t^*, while the target remains the center node *c*^*t*^_*j*_ ∈ *C^t^* of a selected target cluster *j*. The resulting path $P_{u\to c_{j}}^{t}\subseteq G^{t}$ then models an individualized behavioral trajectory from the user’s current position to the target behavioral state at time point *t*. This personalized variant preserves the structural logic of the original graph-based model but enables user-specific transition analysis. To assess its consistency, we compared the aggregated feature transitions derived from this personalized formulation with those obtained under the generalized center-to-center approach.

### High-Variance Features

Following the generation of heatmaps (see step 5, heatmap generation), we analyzed the variance of features along the transition pathways across all behavioral transitions. To focus on meaningful changes during behavioral transitions, our analyses were conducted on high-variance features. These were defined as features that exhibited variance greater than the mean variance across features along the transition paths. Variance was computed for each feature across the users present in each transition path, and features with variance exceeding the mean across all features were classified as high-variance features. However, feature variability can sometimes be specific to individual transitions; that is, certain features may display high variance in one transition but remain stable in others. To ensure that we focus on generalizable patterns of behavior change rather than features relevant to only isolated cases, we selected features that consistently appeared as high variance across multiple transitions. We considered only one direction of each transition between cluster pairs, as the reverse direction would yield the same result. Also, transitions within the same cluster were not considered (7 clusters, yielding 21 unique pairs: cluster 1-cluster 2, cluster 1-cluster 3, etc), because feature behavior is symmetric across both transition directions. GraphBeTraM was developed in Python (version 3.10.12).

### Graph Model Validation

#### Overview

We developed several validation approaches to assess the reliability of the proposed model. First, we evaluated the robustness of the model by conducting a sensitivity analysis of graph construction parameters (k, modularity, and clustering coefficient) and an analysis of graph structure stability and temporal coherence. Second, we evaluated the graph-modeled transitions by assessing (1) the alignment between the transition probabilities derived from GraphBeTraM and the actual transitions observed in the user data, (2) graph temporal dependencies, (3) the impact of missing data in shortest paths, and (4) GraphBeTraM to nongraph baselines. Third, we evaluated the feature transition pathways (1) through personalized shortest paths by assessing the consistency between GraphBeTraM center-to-center feature transition pathways and user-to-center transition pathways, (2) through real-world pathways by assessing the alignment of GraphBeTraM modeled transitions and real-world transitions, and (3) by assessing the stability of features across demographic user subgroups. The methodological details of the validation strategies are outlined below. We defined ρ≥0.90 as very strong, ρ=0.89-0.70 as strong, ρ=0.69-0.40 as moderate, and ρ≤0.39 as weak Spearman correlations. We defined cosine distance ≥0.5 as moderate. We set the significance level to *P*<.05.

#### Validation of Graph Model Construction and Structure

We conducted a sensitivity analysis across k=5 to 20 for all 32 weekly graphs. Euclidean distance was retained as a distance metric because GraphBeTraM operates in a PCA-reduced feature space. We focused on modularity and clustering coefficient as graph-level structural metrics (from the *NetworkX* Python package [version 3.3]), because they capture complementary aspects of graph robustness. Modularity evaluates the stability of global community structure, while the clustering coefficient assesses the stability of local neighborhood cohesion. We also analyzed the structural stability and temporal coherence of GraphBeTraM across the 32 weekly graphs using the graph-level structural metrics (modularity and clustering coefficient) at the selected weekly k values. The value of k was empirically selected for each week to ensure stable neighborhood formation.

#### Validation of Graph Model Transitions

##### Transition Matrix Alignment Between GraphBeTraM and Observed Real-World Transitions

This validation aims to assess the reliability of the constructed graphs by evaluating whether the relative proximity of users (as nodes in the graph) to representative users within clusters (ie, the nodes closest to the k-means centroids) aligns with the users’ observed real-world transitions over time. To be able to quantify this, first we computed a transition matrix from 32-week real user trajectory data by counting transitions between clusters across consecutive weeks and normalizing these counts to obtain transition probabilities. Next, a graph-based transition matrix was derived by assigning each user at each time point to the most probable next cluster based on shortest-path distances to cluster centroids, where distances were converted into probabilities using inverse-distance weighting. These probabilities were used to compute the most likely transitions and aggregated into a transition matrix. Finally, the 2 matrices were compared using Spearman correlation and cosine distance to assess alignment between modeled and observed real-world transitions. Spearman correlation measured agreement in the rank ordering of transition tendencies, whereas cosine distance measured overall vector-level similarity. Further details of the approach can be found in Multimedia Appendix 1.

##### Sensitivity Analysis of Graph Temporal Dependencies

We conducted a sensitivity analysis with dynamic edge reweighting to determine the robustness of GraphBeTraM to temporal dependencies between consecutive weeks. For each week, we did not reconstruct new graphs but instead leveraged the original GraphBeTraM weekly graph topology by reweighting the edges (ie, updating the similarity between users in the same weekly PCA space). We then recomputed the shortest paths and compared weekly transitions with biweekly transitions using Spearman correlation and cosine distance. Further details of the approach can be found in Multimedia Appendix 2.

##### Sensitivity Analysis of Missing Data in Shortest Paths

We conducted a shortest path node-hiding sensitivity analysis to assess the robustness of GraphBeTraM to missing data. For each weekly user-to-centroid shortest path, a fixed proportion of intermediate path nodes (10%, 20%, and 40%) was hidden. The source user node and target centroid node were retained, while one or more intermediate nodes in the path between them were hidden. The user was thus considered disconnected from that specific target centroid, and the disrupted path was excluded from the calculation of transition probabilities. The transition probabilities using inverse shortest-path distances were recalculated across the remaining available original user-to-centroid paths for each user, and the most probable target behavioral state was selected using the argmax function (as described in Multimedia Appendix 1). We assessed the proportion of users who retained at least one user-to-centroid shortest path as a measure of the robustness of GraphBeTraM on the user level. We also measured the alignment between the graph-based transition matrix and the observed real-world transition matrix using Spearman correlation and cosine distance.

##### Transition-Matrix Benchmarking

We conducted a transition modeling comparison analysis against nongraph baselines using the same dataset. Using Spearman correlation and cosine distance, we compared the observed real-world transition matrix with transition matrices from GraphBeTraM and 2 nongraph baselines: (1) a PCA-distance nearest-centroid baseline and (2) an empirical Markov baseline. The PCA-distance nearest-centroid baseline computed the target behavioral state using direct Euclidean distance from each user’s PCA feature vector to behavioral-state centroids. The empirical Markov baseline computed the most frequent next state for each current state based on observed transition frequencies.

### Validation of Feature Transition Pathways

#### Analysis of Personalized Shortest Paths

To validate the feature transition pathway, we focused on validating GraphBeTraM’s ability to capture personalized transitions by comparing user-to-center trajectories to the generalized paths derived from GraphBeTraM center-to-center transitions. In this analysis, we identified users who transitioned from a designated source cluster at Week 0 to a target cluster at Week 31 and compared their 32-week feature evolution to the corresponding model-derived trajectories. This validation method applies GraphBeTraM to users who actually transitioned from a specific cluster to another (eg, from cluster A at Week 0 to cluster B at Week 31). Instead of using the central user node of the source cluster for the transition, we directly consider the actual user nodes and compute their shortest path to the target cluster’s central user node (see Figure 2). This process was repeated for each week. Next, we aggregated the observed pathways across all users having the specified transition over 32 weeks (see Multimedia Appendix 3 for user counts). Since the aggregated pathway across 32 weeks might differ in length from the pathway generated by GraphBeTraM, we aligned them by taking the minimum common path length. Subsequently, we performed correlation and cosine distance computations between the resulting paths and those obtained from GraphBeTraM. We computed Spearman correlation and cosine distance for paths among 42 directed cluster pairs (ie, transitions between 7 clusters in both directions). To obtain an aggregated correlation coefficient for all existing paths between any pair of clusters, we computed the Fisher z score transformed mean [39,40] on the individual Spearman correlation values. Correspondingly, we also computed the mean of the cosine distance scores for these path comparisons.

#### Analysis of Real-World Pathways

To assess the alignment of GraphBeTraM modeled transitions and real-world transitions, we sampled users’ full 32-week trajectories and compressed them to match the length and structure of GraphBeTraM’s paths for direct comparison. This approach, like the previous one, first extracts the user trajectory data with a targeted transition history, considering the label information from the first and last week (eg, trajectories of users who transitioned from cluster A at Week 0 to cluster B at Week 31). Next, we represented 32 weeks of data as rows in a data frame, partitioned it evenly into groups, and calculated the mean of each group to ensure consistency with the resulting path lengths from the GraphBeTraM center-to-center transitions. This validation method does not perform any shortest path analysis. Instead, it relies solely on the original user trajectory data, with aggregation performed for users having the same targeted transitions.

### Stratified Analysis Across Demographic Subgroups

We conducted a stratified analysis to evaluate whether GraphBeTraM’s feature-level pathway validation remains stable across demographic subgroups. Users were stratified by sex, age group, and ethnicity. Age was grouped into 21‐39 years, 40‐59 years, and ≥60 years. Ethnicity was grouped as Chinese and non-Chinese (Indian, Malay, and Others). These groupings were chosen to ensure sufficiently large subgroups for analysis. We compared GraphBeTraM’s modeled pathways with the corresponding observed real-world pathways using Spearman correlation and cosine distance. Spearman correlations were summarized using Fisher z-transformed means. A comparison was considered stable if Spearman ρ≥0.70 and cosine distance ≤0.50. A feature was considered generally stable if it was stable in ≥70% comparisons, partially stable in ≥40%, variable ≥10%, and highly variable if not. Comparisons were made for each transition and demographic subgroup combination with at least 30 users having this transition.

### Analysis of Modeled Transitions

For the analysis of modeled transitions, we focused on potential transitions that reflect substantial behavioral changes such as shifts from inactive to active states. We identified feature-level patterns of change, including their direction, magnitude, and timing, that consistently emerged across users’ behavioral trajectories. By characterizing these transitions (see step 6 feature analysis of transition path), the framework supports the design of targeted nudging strategies that align with users’ natural progression toward healthier behavioral states. We also analyzed the validation results (based on our feature transition pathway validation methods) from the personalized shortest path analysis and real-world feature transition pathways for these transitions to distinguish features that indicate generalizable or context-specific behavioral signals.

We focused on transitions between highly distinct behavioral states that represent meaningful shifts toward healthier behavioral profiles. These transitions are of the greatest value, as they offer the highest potential to inform nudging systems aimed at promoting healthier lifestyles through substantial and consistent behavioral change. We considered five meaningful behavioral state transition scenarios: (1) sedentary office active state to active and healthy eating state, (2) sedentary office active state to highly active state, (3) predominantly sedentary state to highly active state, (4) predominantly sedentary state to active and healthy eating state, and (5) highly active state to active and healthy eating state.

### Ethical Considerations

We conducted this secondary analysis study on digital health application data collected from 36,574 users aged 21 years and older, who were enrolled in a population health program run by the HPB in Singapore. The dataset duration spanned from October 30, 2023, to July 1, 2024. This study was approved by the A*STAR Institutional Review Board (Protocol No. 2023‐080) with a waiver of full informed consent. All data were deidentified before analysis by the study team.

## Results

### Dataset

The demographics of users in the dataset (N=36,574) are presented in Table 3. The mean age of users was 52.9 (SD 12.7) years. There were 22,047 (60.28%) female and 14,527 (39.72%) male users. The majority of users were of Chinese ethnicity 31,069 (84.95%), 1204 (3.29%) were Indian, 504 (1.38%) were Malay, and 3797 (10.38%) were of other ethnicities.

**Table 3.** Demographic characteristics of users in the dataset (N=36,574).

Characteristic	Value
Age in years, mean (SD)	52.9 (12.7)
Sex, n (%)	
Male	14,527 (39.72)
Female	22,047 (60.28)
Ethnicity, n (%)	
Chinese	31,069 (84.95)
Indian	1204 (3.29)
Malay	504 (1.38)
Others	3797 (10.38)

Multimedia Appendix 4 presents the number of users at baseline (October 30, 2023, N=36,574) and demographics of users in 7 behavioral states. Multimedia Appendix 5 shows the mean and median distribution across the 16 behavioral states. Multimedia Appendix 6 shows the distribution of the 16 features across the 7 states.

### High-Variance Features

We computed the high-variance frequency of each feature across the 21 center-to-center cluster pairs. Using the median frequency value of 6 as a threshold, we focused on the top 10 features that were identified as high variance in at least 6 of the 21 transitions. The most frequently identified high-variance features were tracker usage, ratio of sedentary to steps, and weekly moderate to vigorous physical activity (MVPA), each occurring in 15 pairwise transitions (n=15, 71.4%). These were followed by ratio steps to MVPA, last contact, sedentary routine, MVPA on weekends (n=9, 42.9%), and then ratio weekday to weekend, healthy food and drink purchased, and types of exercise classes attended (n=6, 28.6%). This selection ensured that subsequent analyses focused on features with consistent and widespread relevance to behavior change dynamics. Multimedia Appendix 7 shows details of the results.

### Graph Model Validation

#### Validation of Graph Model Construction and Structure

Overall, the k-sensitivity analysis showed a stable graph-level structure across k=5‐20. Modularity was unchanged across k values within each week, with values ranging from 0.666 to 0.703 across weekly graphs. The clustering coefficient remained within a narrow range of approximately 0.256‐0.258. These results suggest that the framework is robust to variations in graph construction parameters. The temporal graph coherence analysis showed that each of the 32 weekly graphs consisted of a single connected structure, confirming that shortest paths were available at each time point. In addition, the graph-level structural metrics remained stable over time. Modularity was consistently high (mean 0.66, SD 0.019; median 0.663, IQR 0.647-0.674) and clustering coefficient showed limited variability (mean 0.269, SD 0.004; median 0.269, IQR 0.265-0.272). These results suggest that although exact edges may vary across independently reconstructed weekly graphs, the higher-level community structure and local connectivity patterns remained consistent across time. Multimedia Appendix 8 shows details of the results.

#### Validation of Graph Model Transitions

##### Transition-Matrix Alignment Between GraphBeTraM and Actual Transitions Observed

A strong Spearman correlation (ρ=0.82; *P*<.001) and low cosine distance (0.05) between the 2 transition matrices (actual and graph-based) supported the validity of the graph-based approach. In particular, strong alignment between observed and modeled transitions supports the hypothesis that real-world behavioral trajectories in digital health programs tend to follow shortest paths toward central, representative behavioral states. Multimedia Appendix 1 shows the actual transition matrix from the dataset and the graph-based transition matrix from GraphBeTraM for the 7 clusters.

##### Sensitivity Analysis of Graph Temporal Dependencies

The sensitivity analysis showed that the temporally edge-reweighted model showed strong alignment with the observed real-world transition matrix, both at the weekly level, with a strong Spearman correlation (ρ=0.74; *P*<.001) and low cosine distance (0.17), as well as at the biweekly level with moderate Spearman correlation (ρ=0.67; *P*<.001) and low cosine distance (0.15). As expected, the temporal edge-reweighted model achieved a lower correlation than GraphBeTraM (see results from 1 above) because it defines user similarity more strictly, because users must be close in the current weekly PCA space and also show similar week-to-week changes. Multimedia Appendix 2 shows details of the results.

##### Sensitivity Analysis of Missing Data in Shortest Paths

The sensitivity analysis showed that GraphBeTraM remained stable when 10% of intermediate nodes were hidden in shortest paths, with 95% (n=34,661) of users retaining available transition probabilities. The graph-based transition matrix remained strongly aligned with the observed real-world transition matrix, with a strong Spearman correlation (ρ=0.78; *P*<.001) and low cosine distance (0.053). When 20% of intermediate nodes were hidden, 17.1% (n=6256) of users retained available transition probabilities, and transition matrix alignment was moderate with Spearman correlation (ρ=0.65; *P*<.001) and low cosine distance (0.07). When 40% of intermediate nodes were hidden, 3.6% (n=1314) of users retained available transition probabilities, with a moderate Spearman correlation (ρ=0.63; *P*<.001) and low cosine distance (0.071). These findings suggest that GraphBeTraM is more susceptible to higher proportions of missing intermediate nodes. Multimedia Appendix 9 shows details of the results.

### Transition-Matrix Benchmarking

GraphBeTraM showed the strongest alignment with the observed real-world transition matrix with a strong Spearman correlation (ρ=0.82; *P*<.001) and low cosine distance (0.05), compared with the 2 nongraph baselines. The PCA-distance nearest-centroid baseline had a moderate Spearman correlation (ρ=0.65; *P*<.001) and low cosine distance (0.06). The empirical Markov baseline also had a moderate Spearman correlation (ρ=0.61; *P*<.001) and low cosine distance (0.05). Multimedia Appendix 10 shows details of the results.

### Validation of Feature Transition Pathways

#### Analysis of Personalized Shortest Paths

Results of the validation of features along the transition pathways for the top 10 high-variance features across 42 transitions are presented in Table 4. We observed strong alignment between the user-to-center and the GraphBeTraM center-to-center transition pathway analyses. Features with high variability (as indicated by SD or IQR), despite having high mean correlation values, may reflect context-specific behavior; for example, they may show strong alignment in certain transitions but weaker alignment in others. This variability may also have been influenced by the uneven distribution of actual user transitions across cluster pairs (ranging from 11 to 1528 users), which may affect the stability of correlation estimates. Nonetheless, most features showed strong mean correlation scores (≥0.70) and low mean cosine distance scores (≤0.49). In particular, for key physical activity features, including ratio sedentary to steps with a very strong mean correlation (mean 0.99, SD 0.96) and low mean cosine distance (mean 0.20, SD 0.28), as well as Weekly MVPA with a very strong mean correlation (mean 0.97, SD 0.96) and low mean cosine distance (mean 0.24, SD 0.35). However, healthy food and drink purchased shows a moderate mean correlation (mean 0.58, SD 0.53) despite a low cosine distance (mean 0.12, SD 0.30), which may indicate that the relative positioning (ie, rank order) of the feature values differs, but their overall direction aligns. Conversely, the sedentary routine showed a strong mean correlation (mean 0.83, SD 0.95) but only a moderate cosine distance (mean 0.53, SD 0.58). This suggests that while the overall vector directions differ, the feature maintains a robust rank-based relationship, indicating that patterns of value change and relative ordering were well aligned across transitions.

**Table 4.** Validation results for top the 10 high-variance features, showing Fisher z-transformation mean Spearman correlation and mean cosine distance scores across 42 transitions.

Features	Spearman ρ (n=42 transitions)		Cosine distance (n=42 transitions)	
	Mean (SD)	Median (IQR)	Mean (SD)	Median (IQR)
Tracker usage	0.69 (0.80)	0.73 (0.34 to 0.91)	0.36 (0.40)	0.17 (0.10 to 0.61)
Ratio sedentary to steps	0.99 (0.96)	0.96 (0.05 to 1.00)	0.20 (0.28)	0.08 (0.03 to 0.24)
Weekly MVPA^a^	0.97 (0.96)	0.93 (–0.35 to 1.00)	0.24 (0.35)	0.08 (0.04 to 0.41)
Ratio steps to MVPA	0.89 (0.98)	0.75 (–0.05 to 0.96)	0.46 (0.57)	0.17 (0.07 to 0.80)
Last contact	0.84 (0.90)	0.78 (0.27 to 0.95)	0.32 (0.34)	0.16 (0.06 to 0.58)
Sedentary routine	0.83 (0.95)	0.75 (0.14 to 0.95)	0.53 (0.58)	0.24 (0.11 to 0.68)
MVPA on weekends	0.81 (0.97)	0.83 (0.36 to 0.96)	0.36 (0.34)	0.24 (0.10 to 0.57)
Ratio weekday to weekend	0.85 (0.94)	0.58 (–0.01 to 0.88)	0.42 (0.28)	0.39 (0.23 to 0.55)
Healthy food and drink purchased	0.58 (0.53)	0.61 (0.31 to 0.80)	0.12 (0.30)	0.01 (0.00 to 0.04)
Types of exercise classes attended	0.83 (0.53)	0.80 (0.62 to 0.90)	0.09 (0.21)	0.00 (0.00 to 0.05)

a MVPA: moderate to vigorous physical activity.

#### Analysis of Real-World Pathways

We computed Spearman correlation and cosine distance scores between the real user paths and those generated by GraphBeTraM aggregated over the top 10 high-variance features across 42 transitions (for details, see Model Validation Methods). We present the results in Table 5. We observed that overall, GraphBeTraM captured key patterns from the real user 32-week data, especially for key features such as ratio sedentary to steps with a strong mean correlation (mean 0.79, SD 0.79) and low mean cosine distance (mean 0.39, SD 0.28), as well as weekly MVPA with a strong mean correlation (mean 0.79, SD 0.81) and low mean cosine distance (mean 0.41, SD 0.39). However, MVPA on weekends shows a weak mean correlation (mean 0.24, SD 0.61) and moderate cosine distance (mean 0.83, SD 0.41). In addition, ratio weekday to weekend showed a weak mean correlation (mean 0.28, SD 0.72) and moderate cosine distance (mean 0.93, SD 0.33), as well as healthy food and drink purchased, with a weak mean correlation (mean 0.26, SD 0.77) and moderate cosine distance (ρ=0.77, SD 0.44). This may indicate that features related to personal preferences, such as time constraints, (like MVPA on weekends and weekday to weekend) or related to purchase or engagement (like healthy food, drink purchased, and last contact) exhibit context-specific relevance, while physical activity features like ratio sedentary to steps and weekly MVPA usually emerged as robust, generalizable indicators of change.

**Table 5.** Real-world pathways Validation results for top the 10 high-variance features, showing Fisher z-transformation mean Spearman correlation and mean cosine distance scores across 42 transitions.

Features	Spearman ρ (n=42 transitions)		Cosine distance (n=42 transitions)	
	Mean (SD)	Median (IQR)	Mean (SD)	Median (IQR)
Tracker usage	0.61 (0.55)	0.63 (0.19 to 0.82)	0.43 (0.30)	0.37 (0.19 to 0.57)
Ratio sedentary to steps	0.79 (0.79)	0.73 (0.43 to 0.88)	0.39 (0.28)	0.32 (0.18 to 0.55)
Weekly MVPA^a^	0.79 (0.81)	0.70 (0.43 to 0.89)	0.41 (0.39)	0.26 (0.15 to 0.50)
Ratio steps to MVPA	0.62 (0.75)	0.51 (0.08 to 0.79)	0.54 (0.43)	0.40 (0.16 to 0.81)
Last contact	0.41 (0.58)	0.34 (–0.08 to 0.71)	0.65 (0.34)	0.60 (0.42 to 0.90)
Sedentary routine	0.64 (0.82)	0.44 (0.15 to 0.76)	0.62 (0.44)	0.56 (0.23 to 0.87)
MVPA on weekends	0.24 (0.61)	0.17 (–0.20 to 0.62)	0.83 (0.41)	0.86 (0.48 to 1.03)
Ratio weekday to weekend	0.28 (0.72)	0.12 (–0.21 to 0.51)	0.93 (0.33)	0.91 (0.77-1.08)
Healthy food and drink purchased	0.26 (0.77)	0.08 (–0.44 to 0.54)	0.77 (0.44)	0.87 (0.42 to 1.11)
Types of exercise classes attended	0.50 (0.60)	0.58 (0.13 to 0.76)	0.68 (0.40)	0.66 (0.39 to 1.00)

a MVPA: moderate to vigorous physical activity.

We note that the above scores were computed across all 42 transitions (listed in Multimedia Appendix 3), including those between indistinct behavioral states with comparable underlying profiles such as similar levels of MVPA or steps. However, this study focused on analyzing how users change over time between different behavioral patterns, with insights that may inform nudging approaches based on factors such as timing (eg, early vs later), duration (eg, weekly vs longer periods), and the specific aspects to target (eg, physical activity vs engagement). Thus, we further analyzed these transitions by looking at transitions between highly distinct behavioral states in the following section, Analysis of Modeled Transitions.

### Stratified Analysis Across Demographic Subgroups

We focused the analysis on the 5 behavioral state transition scenarios examined in the analysis of modeled transitions. We identified 23 transition-subgroup combinations with at least 30 users having these transitions. Weekly MVPA was the only feature classified as broadly generalizable, meeting the stability criterion in 21 (91%) comparisons with a strong mean Spearman correlation (mean 0.98, SD 2.36) and low mean cosine distance (mean 0.23, SD 0.27). This indicated that weekly MVPA remained consistently aligned with the modeled pathways across age, sex, and ethnicity. Other features showed more transition- or subgroup-specific stability. Healthy food and drink purchased met the stability criterion in 61% (n=14) of comparisons, with a strong mean Spearman correlation (mean 0.93, SD 1.69) and low-to-moderate mean cosine distance (mean 0.40, SD 0.39). These stable transition-subgroup comparisons occurred most often in transitions toward the active and healthy eating state. This suggested that healthy food and drink purchasing behavior was consistently captured across age, sex, and ethnicity when the target state was directly related to healthier eating behavior. Healthy food and drink purchased appeared to be a transition-relevant and subgroup-consistent feature for eating-related transitions, rather than a generalizable feature across all behavioral transitions. Ratio of sedentary to steps and last contact each met the stability criterion in 52% (n=12) of comparisons, suggesting partial stability. In contrast, time-related features showed high variability across subgroups; MVPA on weekends and the ratio of weekday to weekend were stable in only 4% (n=1) of comparisons. These findings support the interpretation of weekly MVPA as a stable generalizable signal of behavioral change across demographic subgroups, while other features may be more context-specific. Particularly, healthy food and drink purchased shows more stability when the transition end point is relevant to healthy eating behavior, whereas engagement and time preference features appeared more subgroup- or transition-dependent. Multimedia Appendix 11 shows details of the results.

### Analysis of Modeled Transitions

We further analyzed the outcomes of GraphBeTraM by focusing on the modeled transitions between highly distinct behavioral states for the 5 selected scenarios outlined in Methods. As expected, some transitions had few users who made these transitions in the real world, while other transitions have a lot more users. For example, for a rather extreme transition from predominantly sedentary to highly active, there are only 62 users with this actual transition in the dataset, whereas for a more common transition like sedentary office active to very active, there are 1055 users. Multimedia Appendix 1 lists for all 42 scenarios the number of users who transitioned from a source cluster in Week 0 to a target cluster in Week 31, capturing a 32-week transition period. In the following, we present transition scenario 1, sedentary office active to active and healthy eating, as this is a very relevant scenario for many users in sedentary jobs.

### Sedentary Office Active to Active and Healthy Eating (n=646)

**Figure 3.**
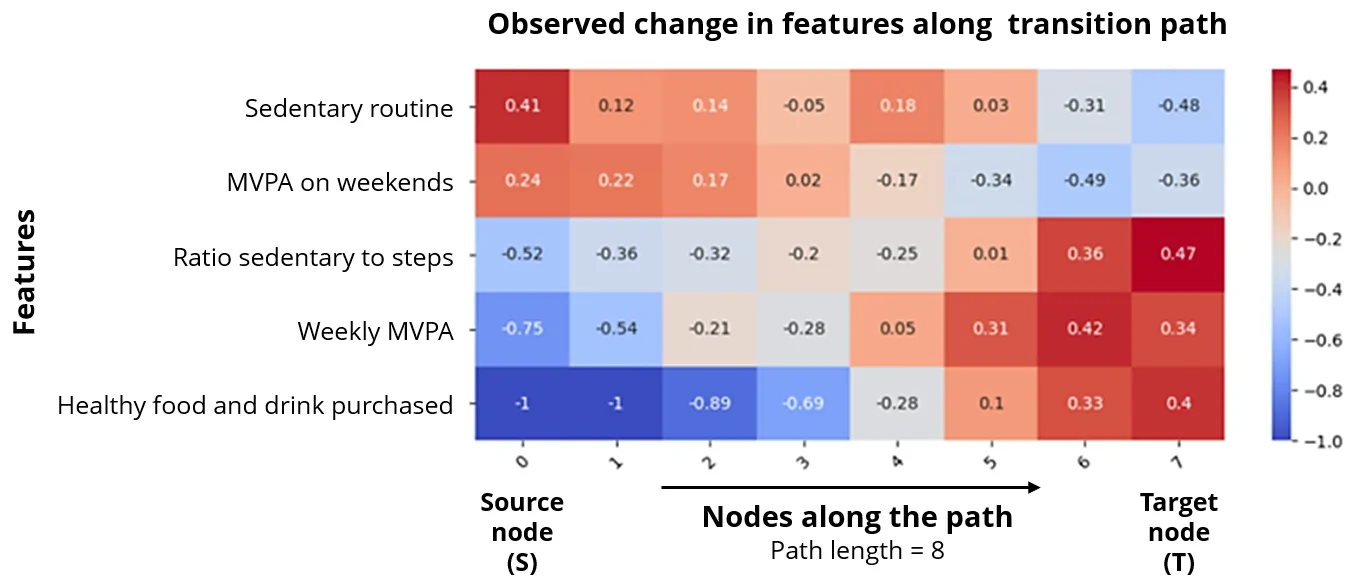
Outcome heatmap from the graph-based behavioral trajectory model (GraphBeTraM) for the transition from the sedentary office-active state to the active and healthy eating state, showing the top 5 high-variance features to observe changes along the path from source node (S) to target node (T). MVPA: medium to vigorous physical activity.

The analysis of this transition revealed nuanced shifts across key high-variance behavioral features. In Figure 3, we present the GraphBeTraM output heatmap showing feature changes along the transition pathway from the source node (S) in the sedentary office active state to the target node (T) in the active and healthy eating state. The path had a length of 8 nodes, with each node representing a behavioral feature vector. We focused on the top 5 high-variance behavioral features for this transition, including sedentary routine, MVPA on weekends, ratio sedentary to steps, weekly MVPA, and healthy food and drink purchased. The heatmap showed how these 5 features evolve along the path from (S) to (T). The top 2 rows in the heatmap showed a decrease in sedentary routine from step 0 (S=0.41) to step 7 (T=−0.48), as well as a decrease in MVPA on weekends (S=0.24 to T=−0.36). While the lower 3 rows showed an increase in ratio sedentary to steps (S=−0.52 to T=0.47), weekly MVPA (S=−0.75 to T=0.35), and healthy food and drink purchased (S=−1 to T=0.4).

**Figure 4.**
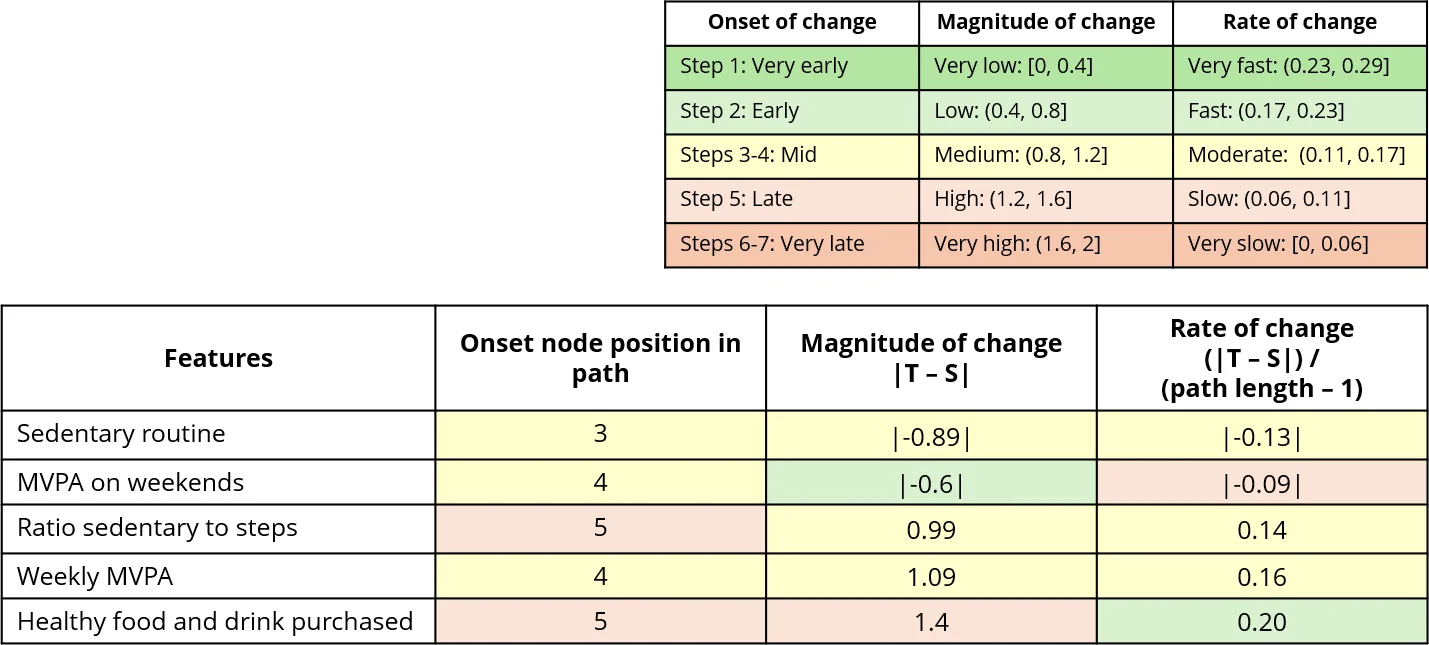
Outcome of graph-based behavioral trajectory model (GraphBeTraM) for transition from sedentary office active state to active and healthy eating state, showing thresholds for onset, magnitude, and rate of change and a summary table of onset node position in path, magnitude of change, and rate of change for the top 5 high-variance features. MVPA: medium to vigorous physical activity; S: source node; T: target node.

In Figure 4, we present a summary of these 5 key features, showing their onset, magnitude, and RoC along the transition pathway. For this transition, the thresholds for onset of change were determined as: step 1 (very early) to step 6‐7 (very late); magnitude of change as very low (0-0.4) to very high (1.6-2); and RoC as very fast (0.23-0.29) to very slow (0-0.06). Onset node positions in the path ranged from step 3: mid onset (sedentary routine) to step 5: late onset (ratio sedentary to steps and healthy food and drink purchased), indicating that these 2 features may be either more resistant to change or are more characteristic of the sedentary office active state and active and healthy eating state, respectively. The magnitude of change, calculated as |T - S|, ranged from Low: 0.6 (MVPA on weekends) to High: 1.4 (healthy food and drink purchased), indicating that the 2 states were closer in physical activity (MVPA on weekends) than they are in purchasing healthy food or drinks. The RoC, calculated as (|T – S|)/(path length – 1) ranged from Fast: 0.20 (healthy food and drink purchased) to Slow: 0.09 (MVPA on weekends), indicating a potentially rapid change in purchasing behavior, but a slower change in time preferences for physical activity (MVPA) on weekends.

In Table 6, we analyzed both the personalized shortest pathways and the real-world pathways for this transition to understand the features that are generalizable and context-specific signals. Both weekly MVPA with a very strong Spearman correlation (ρ_personalized_=0.94; *P*<.01; ρ_real-world_=0.90; *P*<.001), and low cosine distance (0.11; 0.12), as well as healthy food and drink purchased with a very strong Spearman correlation (ρ_personalized_=0.90; *P*=.01; ρ_real-world_=0.97; *P*<.001), and low cosine distance (0.02; 0.12), were validated by both pathway validation methods, suggesting that they are strong signals for this transition. This was expected because the active and healthy eating state represents a more engaged and physically active behavioral state compared to the sedentary office active state. The target state also reflected greater interest in purchasing healthy food and drink. In contrast, sedentary routine, MVPA on weekends, and ratio sedentary to steps, though considered high-variance features, appeared to be context-specific and were not consistently valid across users.

**Table 6.** Validation results of the feature transition pathway (personalized shortest pathways and real-world pathways) showing Spearman correlation and cosine distance scores for the transition between the sedentary office active state and the active and healthy eating state (n=646).

Features	Personalized shortest pathways			Real-World pathways		
	Spearman ρ	*P* value	Cosine distance	Spearman ρ	*P* value	Cosine distance
Sedentary routine	0.49	.33	0.31	0.45	.26	0.49
MVPA^a^ on weekends	1.00	<.001	0.21	0.48	.23	0.42
Ratio sedentary to steps	0.94	<.01	0.23	0.38	.35	0.68
Weekly MVPA	0.94	<.01	0.11	0.90	<.001	0.12
Healthy food and drink purchased	0.90	.01	0.02	0.97	<.001	0.12

a MVPA: moderate to vigorous physical activity.

From these observations, we can derive insights on how to nudge users in the sedentary office active state to transition to the active and healthy eating state. For example, the feature, healthy food and drink purchased, showed the highest magnitude and the fastest RoC, indicating that once initiated, changes in dietary behavior may occur rapidly, even if they do emerge later in the behavior change trajectory. In contrast, MVPA on weekends changed slowly and with low magnitude, perhaps indicating more reluctance to changing time preferences.

Furthermore, we observed that the feature ratio sedentary to steps, which reflects the balance between sedentary duration and step count, showed a late onset to change (step 5: late), a medium magnitude of change (medium: 0.99), and a moderate RoC (moderate: 0.14). This change in time preference would probably be hard to achieve for users in the sedentary office active state, because this state tends to show more sedentary behavior during morning hours, likely reflecting time-dependent inactivity due to inflexible work schedules or other structured commitments. In contrast, the active and healthy eating state exhibits lower total sedentary time, which may indicate more flexible schedules or increased awareness and intentional activity planning. We also observed an early onset of change (step 3: early), a medium magnitude of change (medium: 0.89), and a fast RoC (fast: 0.13) in the sedentary routine, which is a feature for tracking overall inactivity throughout the day. This pattern suggests that reducing overall sedentary time could be an achievable early milestone, particularly if users are supported to break up long periods of inactivity with brief movements. Overall, these insights highlight the importance of addressing both temporal and inactivity patterns to support behavior change. For example, interventions could target inactivity early with light movement nudges and introduce healthy food choices later but persistently.

Table 7 summarizes the feature-level transition dynamics identified by GraphBeTraM for the transition between the sedentary office active state and the active and healthy eating state. Each row represents a dimension of a feature-level transition dynamic, such as the rate, magnitude, and timing of change, and maps it to its corresponding intervention insight that informs nudging strategies. Behavioral features are categorized according to whether (for this transition) they have been identified as generalizable signals (weekly MVPA and healthy food and drink purchased) and suitable for population-level intervention or as context-specific signals (sedentary routine, MVPA on weekends, and ratio sedentary to steps), which are better delivered as personalized interventions. They are further grouped by when they are expected to change along the trajectory (early vs later stages), this informs when they should be delivered during the transition intervention. It might be more encouraging to start with some quick wins and then introduce the more difficult changes. The magnitude of change gives guidance as to the amount of effort that would be needed to make this change, and therefore the intensity of the intervention can be planned accordingly, if daily or weekly nudges are appropriate. Also, the expected RoC informs how long the delivery cycle needs to be; if a change is expected to be fast, then a short, intense intervention might be suitable, whereas if the change is expected to be slow, then a more gentle but persistent approach might be more effective. Finally, these feature-level dynamics and their corresponding intervention insights are mapped to possible nudging strategies that incorporate these insights to design appropriate push notifications, goal-setting prompts, or recommendations. We note that GraphBeTraM does not implement nor evaluate specific intervention strategies but rather provides a framework that informs when and how interventions could be most effectively designed based on data-driven feature-level dynamics.

**Table 7.** Graph-based behavioral trajectory model (GraphBeTraM) behavioral dynamics translated into actionable nudging strategies for the transition between the sedentary office active state and the active and healthy eating state.

Feature-level transition dynamics → informs intervention design component	Sedentary routine	MVPA^a^ on weekends	Weekly MVPA	Ratio sedentary to steps	Healthy food and drink purchased
Transition signal strength → type of intervention	Context-specific signal (weaker and less consistent) → personalized intervention	Context-specific signal (weaker and less consistent) → personalized intervention	Generalizable signal (stronger, consistent) → population-level intervention	Context-specific signal (weaker and less consistent) → personalized intervention	Generalizable signal (stronger, consistent) → population-level intervention
Expected onset of change in trajectory → timing of delivery	Expected mid-onset → deliver in early-to-mid stage of intervention	Expected mid-onset → deliver in early-to-mid stage of intervention	Expected midonset → deliver in early-to-mid stage of intervention	Expected late change → deliver in later stage of intervention	Expected late change → deliver in later stage of intervention
Expected magnitude of change (effort needed for change) → intensity of intervention	Moderate effort → moderate intensity (eg, nudges every 2‐3 d)	Low effort → low intensity (eg, weekly nudges)	Moderate effort → moderate intensity (eg, nudges every 2‐3 d)	Moderate effort → moderate intensity (eg, nudges every 2‐3 d)	High effort → high intensity (eg, daily nudges)
Expected rate of change → duration of intervention	Moderate → moderate-length intervention	Slow → longer intervention	Moderate → moderate-length intervention	Moderate → moderate-length intervention	Fast → shorter intervention
Possible nudge strategy	Personalized, moderate nudging for example, reminders to break sedentary time	Personalized, gentle but persistent nudging to start MVPA on weekends	General moderate nudging to increase overall weekly MVPA	Personalized, moderate nudging, for example, reminders during lunch break to go for a brisk walk	Short, high-intensity intervention, for example, prompts to try healthy food options

a MVPA: moderate to vigorous physical activity.

Analyses of transition scenarios (2) sedentary office active state to highly active state (n=1055), (3) predominantly sedentary state to highly active state (n=62), (4) predominantly sedentary state to active and healthy eating state (n=86), and (5) highly active state to active and healthy eating state (n=443) can be found in Multimedia Appendices 12-15.

## Discussion

### Principal Findings

This study presents a graph-based behavioral trajectory modeling framework, GraphBeTram, which is designed to characterize user transitions in longitudinal digital health data. The proposed framework enables a structured and interpretable analysis of behavior change trajectories by modeling transitions as shortest paths through user similarity graphs.

Heatmap visualizations revealed progressive patterns of change across features. The approach was further supported by validation against observed and modeled transition matrices and pathways. Physical activity markers, such as weekly MVPA, were found to be reliable indicators by showing steady and validated changes across multiple pathways (see transition scenarios 2, 3, 4, and 5). These features aligned with observed real-world behavior and evolved gradually, suggesting that they may be broadly applicable for monitoring progression and informing the design of future digital health interventions. In contrast, features tied to personal preferences, including time constraints or engagement such as MVPA on weekends (see transition scenarios 1, 3, and 4) and healthy food and drink purchased (see transition scenarios 1, 4, and 5), showed context-specific relevance that varied across transitions. Together, these findings support both personalized and population-level analyses and distinguish stable, generalizable patterns from context-specific patterns.

We also observed a distinction between features that may correspond to behavioral state markers versus those that may serve as dynamic change signals. For example, last contact effectively distinguished between clusters but remained stable throughout transitions, indicating that some engagement-related features may reflect user states rather than gradual behavioral change. Furthermore, validation of both personalized and generalized pathways revealed strong consistency between individualized and center-to-center models. A stratified analysis across demographics further supports the distinction between generalizable and context-specific features. Generalizable features, particularly those related to physical activity (weekly MVPA), remained stable across demographic subgroups, reinforcing their utility as population-level indicators of behavioral change. In contrast, context-specific features such as healthy food and drink purchased varied more substantially across subgroups, suggesting that these features may reflect differences in personal preferences such as time constraints. By integrating relational structure with feature-level dynamics, the framework quantifies the direction, magnitude, and timing of change and thus provides deeper insight into the dynamics of behavior change within real-world digital health programs.

Overall, these findings demonstrate how GraphBeTraM could inform the design of digital health interventions by aligning nudges with the natural progression of behavior change, prioritizing features that tend to change early as quick wins, while sustaining engagement for behaviors that require longer periods to shift.

### Comparison With Prior Work

By modeling behavioral transitions as shortest paths within user similarity graphs where central cluster nodes anchor transitions and enable detection of nuanced shifts, GraphBeTraM overcomes the reliance on rigid parametric assumptions, which can be a limitation of model-based trajectory modeling techniques, such as latent class growth analysis, growth mixture modeling, and group-based trajectory modeling [26-30]. Additionally, GraphBeTraM explicitly represents relations among states, quantifies direction, magnitude, and timing of change, and improves temporal and contextual sensitivity. Thus, it addresses a key limitation of distance-based trajectory clustering methods, such as those based on Euclidean distance [31-33] or dynamic time warping [34,35], which do not capture transitions between behavioral states and their relational structure. Furthermore, GraphBeTraM dynamically captures the progression toward behavioral states by aggregating shortest-path trajectories across weeks and thus addresses the limited focus on short-term behavior of many sequence and pattern mining approaches [36,37]. Finally, behavior modeling approaches often represent behavior as a sequential and memoryless progression through discrete “states” [23,30,33,37], without considering dependencies and contextual factors such as time of day or social settings. Graph-based models address this limitation by explicitly capturing relational structure among states and transitions [41-43]. This work contributes to the digital health and behavior change modeling literature by integrating both graph-based structural modeling and feature-level behavioral dynamics.

### Limitations and Future Directions

In this study, we focused our analysis on users with complete longitudinal data to ensure consistency in trajectory modeling and interpretation. As such, the dataset did not contain missing data, and no imputation nor formal testing for missing data completely at random was performed. Although the underlying data collection process did include an adjudication window that allowed delayed capture of wearable data following synchronization failures, this does not eliminate missingness completely. Thus, the framework might be limited in its applicability in real-world digital applications where missing data are common due to unsynchronized devices or incomplete behavioral logging. Future work should explore data imputation and graph-based methods that accommodate missing data. Also, practical thresholds for data completeness should be determined to improve real-world deployment value.

Furthermore, the framework assumes behavior change occurs as a gradual shift between similar behavioral states. This, however, may not hold true in real-world settings where users could display abrupt transitions or follow multiple alternative paths. User similarity graphs and shortest paths are computed independently at each time point, and resulting pathways are aggregated across weeks to smoothen local variations or short-term fluctuations (eg, temporary interventions or seasonal effects); thereby, temporal dynamics are captured through the accumulation of transitions over time. However, the framework does not explicitly model temporal dependencies nor temporal consistency in graph structure; thus, changes in graph topology may affect the stability and interpretation of transition pathways. In addition, although onset timing and rate of change provide useful temporal insights, they might not capture more complex dependencies. Overall, GraphBeTraM captures interpretable transition patterns at the population level but does not aim to reproduce explicit individual pathways. Future work could address this limitation by using dynamic graph structures for richer temporal modeling.

Finally, our validation strategy mainly assessed the internal consistency of GraphBeTraM by comparing its modeled transitions with observed real-world transitions and examining its robustness to graph construction, structure, and feature transition pathways. This validation showed that GraphBeTraM aligns closely with real-world transitions and captures meaningful behavioral dynamics. We also performed an initial comparison with 2 nongraph baselines; however, this did not aim to be a comprehensive benchmarking. Existing approaches assume different constraints and do not explicitly model transitions nor feature-level trajectories like GraphBeTraM does, thus making quantitative comparisons challenging and beyond the scope of this study. Future work could develop a benchmarking framework to empirically compare complementary approaches, including graph approaches using different distance metrics, as well as nongraph methods such as latent class growth analysis or dynamic time warping clustering.

### Implications

The main contribution of this work lies in establishing a framework that models behavior change not as isolated events but as dynamic sequences within a structured behavioral state space. By capturing the temporal evolution of behavioral states, the framework enables a richer and more realistic representation of behavior change. A key strength of the proposed approach lies in its ability to distinguish between generalizable patterns and context-specific features, offering a more nuanced understanding of behavioral change dynamics. Furthermore, its flexibility in analyzing both population-level trends as well as individual-level trajectories positions it as a valuable tool for the retrospective evaluation of behavioral patterns, as well as to inform the design of forward-looking, adaptive interventions.

### Conclusion

This study explored the utility of GraphBeTraM, a graph-based framework for modeling user behavioral transitions in a digital health application. By quantifying direction, timing, and magnitude of change along shortest-path trajectories, the model captures user progression in a structured and interpretable manner. Validation against real-world transition patterns demonstrated strong alignment, underscoring the suitability of graph-based representations for characterizing behavior change. This framework offers a deeper understanding of behavior change in real-world settings to support the design of digital health interventions that align with users’ natural progression toward healthier behaviors.

## Supplementary material

10.2196/86629 Multimedia Appendix 1 Transition-matrix alignment between graph-based behavioral trajectory model (GraphBeTraM) and actual transitions observed.

10.2196/86629 Multimedia Appendix 2 Sensitivity analysis of graph temporal dependencies.

10.2196/86629 Multimedia Appendix 3 Number of users transitioning from source state or cluster in Week 0 to target state or cluster in Week 31. To model a 32-week behavioral transition period for 42 state or cluster pairs: MVPA-focused active, step-focused active, predominantly sedentary, highly active, sedentary office active, active and healthy eating, and class-based active.

10.2196/86629 Multimedia Appendix 4 Number of users at baseline (October 30, 2023; N=36,574) and demographics of users in 7 behavioral states.

10.2196/86629 Multimedia Appendix 5 Behavioral features in the dataset showing mean and median distributions across users (N=36,574) in the dataset.

10.2196/86629 Multimedia Appendix 6 Distribution of average feature values across users in 7 behavioral states (N=36,574).

10.2196/86629 Multimedia Appendix 7 Features and their frequency as high-variance features across 21 pairwise transitions formed from 7 behavioral states.

10.2196/86629 Multimedia Appendix 8 Validation of graph model construction and structure.

10.2196/86629 Multimedia Appendix 9 Sensitivity analysis of missing data in shortest paths.

10.2196/86629 Multimedia Appendix 10 Transition-matrix benchmarking against nongraph baselines.

10.2196/86629 Multimedia Appendix 11 Stratified analysis by demographic characteristics.

10.2196/86629 Multimedia Appendix 12 Transition scenario B: sedentary office active to highly active.

10.2196/86629 Multimedia Appendix 13 Transition scenario C: predominantly sedentary to highly active.

10.2196/86629 Multimedia Appendix 14 Transition scenario D: predominantly sedentary to active and healthy eating.

10.2196/86629 Multimedia Appendix 15 Transition scenario E: highly active to active and healthy eating.

## Abbreviations

GraphBeTraM: graph-based behavioral trajectory model
HPB: Health Promotion Board
k-NN: k-nearest neighbor
MVPA: medium to vigorous physical activity
PCA: principal component analysis
RoC: rate of change
