# Supplementary material for: Graph-Based Modeling of Behavioral Transitions for Digital Health Applications: Trajectory Analysis Framework
Source: J Med Internet Res. 2026 Sep 4;28:e86629. doi: 10.2196/86629 (PMC13544619; doi:10.2196/86629)
Supplement: Supplementary file 1 [file jmir-v28-e86629-s001.docx]

## **Multimedia Appendix 1.** Transition-matrix alignment between GraphBeTraM and actual transitions observed

The transition matrix is constructed by counting the frequency with which users move from one cluster to another across consecutive timepoints as shown below, followed by row-wise normalization to obtain a probability distribution representing the likelihood of transitioning between clusters over time.

$$M_{i,j}=\sum_{u} \sum_{t=1}^{T-1} I\left( C_{t}^{u}=i\wedge C_{t+1}^{u}=j \right)\quad\left( 1 \right)$$

where:

- $M_{i,j}$ is the number of observed transitions from cluster *i* to cluster *j*
- $C_{t}^{u}$is the cluster assignment of user *u* at timepoint *t*
- $I(.)$ is the indicator function (1 if the condition is true, 0 otherwise)
- *T* is the total number of timepoints

Then a graph-based transition matrix is computed that captures the association between a user’s current cluster at timepoint *t* and the cluster they are most likely to transition to at timepoint *t* + 1, based on graph-derived transition probabilities. For each user and timepoint, we determine the most probable next cluster by identifying the centroid with the highest transition probability ${\text{probable}\text{\_}\text{cluster}}_{t}=\arg\max_{i} P_{i}^{t}$. We then update the matrix by incrementing the count for the corresponding cluster pair ($C_{t}^{u},{\text{probable}\text{\_}\text{cluster}}_{t})$.

To calculate these time-specific transition probabilities, the model first computes the shortest path distance $d_{i}^{t}=\text{ShortestPathDistance}\left( u^{t},\text{centroid}_{i}^{t} \right)$, from the user node at timepoint *t*, denoted $u^{t}$, to each cluster centroid at that timepoint. Here, shortest path distance is defined as the sum of edge weights along the path, where each edge weight corresponds to the Euclidean distance between PCA-transformed user vectors, as determined during k-NN-based graph construction. To emphasize proximity, each distance is then transformed into its inverse $\text{inv}_{i}^{t}=\frac{1}{d_{i}^{t}+\epsilon}$, where ϵ is a small constant for numerical stability. These inverse values are then normalized to produce a valid probability distribution for timepoint *t*, $P_{i}^{t}=\frac{\text{inv}_{i}^{t}}{\sum_{j=1}^{K} \text{inv}_{j}^{t}}$ , where $K$ is the number of clusters, $P_{i}^{t}$ is the probability of user *u* transitioning to cluster *i* at timepoint *t* + 1.

The resulting graph-based transition matrix, $M_{i,j}^{\text{graph}}=\sum_{u} \sum_{t=1}^{T-1} I\left( C_{t}^{u}=i\wedge{\text{probable}\text{\_}\text{cluster}}_{t}^{u}=j \right)$, captures the most likely temporal transitions, providing a time-sensitive view of how users are expected to evolve across behavioral clusters over time. Similar to the observed transition matrix $M_{i,j}$, we applied row-wise normalization to $M_{i,j}^{\text{graph}}$and compared the two by computing Spearman correlation and cosine distance scores between their flattened vector representations.

Table 1.1 shows the results for the actual transition matrix from the dataset and Table 8.2 shows the graph-based transition matrix from GraphBeTraM for the 7 clusters.

**Table 1.1.** Actual transition matrix derived from the original data for the 7 state/cluster pairs.

|  | MVPA-focused Active | Step-focused active | Predominantly sedentary | Highly active | Sedentary office active | Active and healthy eating | Class-based active |
| --- | --- | --- | --- | --- | --- | --- | --- |
| MVPA-focused active | 84.21 | 3.64 | 0.27 | 4.94 | 5.56 | 1.17 | 0.21 |
| Step-focused active | 18.81 | 38.97 | 8.09 | 0.39 | 32.97 | 0.7 | 0.08 |
| Predominantly sedentary | 1.62 | 3.91 | 64.31 | 0.59 | 28.24 | 1.11 | 0.21 |
| Highly active | 4.38 | 0.15 | 0.15 | 88.51 | 5.11 | 1.49 | 0.19 |
| Sedentary office active | 2.59 | 3.42 | 3.03 | 2.47 | 86.95 | 1.3 | 0.25 |
| Active and healthy eating | 3.27 | 0.67 | 0.83 | 4.36 | 8.05 | 82.54 | 0.28 |
| Class-based active | 1.45 | 0.22 | 0.39 | 1.5 | 3.75 | 0.68 | 92.01 |

**Table 1.2.** Graph-based transition matrix for 7 state/cluster pairs.

|  | MVPA-focused Active | Step-focused active | Predominantly sedentary | Highly active | Sedentary office active | Active and healthy eating | Class-based active |
| --- | --- | --- | --- | --- | --- | --- | --- |
| MVPA-focused active | 49.15 | 4.17 | 1.17 | 24.38 | 12.15 | 8.38 | 0.60 |
| Step-focused active | 12.38 | 65.92 | 3.76 | 0.15 | 15.91 | 1.82 | 0.05 |
| Predominantly sedentary | 2.12 | 2.18 | 82.48 | 1.38 | 10.03 | 1.65 | 0.15 |
| Highly active | 11.7 | 0.34 | 1.27 | 71.17 | 7.68 | 7.15 | 0.68 |
| Sedentary office active | 9.76 | 6.09 | 1.49 | 7.28 | 65.41 | 9.68 | 0.29 |
| Active and healthy eating | 4.21 | 1.95 | 1.65 | 3.92 | 4.49 | 82.98 | 0.79 |
| Class-based active | 0.06 | 0.04 | 0.29 | 0.01 | 0.03 | 0.20 | 99.37 |
