# Supplementary material for: Graph-Based Modeling of Behavioral Transitions for Digital Health Applications: Trajectory Analysis Framework
Source: J Med Internet Res. 2026 Sep 4;28:e86629. doi: 10.2196/86629 (PMC13544619; doi:10.2196/86629)
Supplement: Supplementary file 2 [file jmir-v28-e86629-s002.docx]

## **Multimedia Appendix 2.** Sensitivity Analysis of Graph Temporal Dependencies

We conducted a temporal sensitivity analysis of GraphBeTraM using dynamic edge reweighting and temporal granularity comparison. The temporal edge-reweighted model preserves the saved weekly graph topology and recomputes shortest paths with edge weights that combine the original current-week PCA distance with week-to-week PCA change similarity.

For each edge between users *i* and *j* at week *t*, the temporally reweighted edge weight was defined as:

$$w_{ij}^{t,\mathrm{dyn}}=w_{ij}^{t}+{\parallel\Delta z_{i}^{t}-\Delta z_{j}^{t}\parallel}_{2}$$

where $w_{ij}^{t}$is the original edge weight in the saved week-$t$ graph, which corresponds to the current-week Euclidean distance between the PCA representations of users *i* and *j*. The PCA representation of user *i* at week *t* is denoted by $z_{i}^{t}$, and the week-to-week change vector is defined as:

$$\Delta z_{i}^{t}=z_{i}^{t}-z_{i}^{t-1}$$

The second term measures how different the recent behavioral changes of users *i* and *j* were between week *t* – 1 and week *t*. Thus, the temporal edge-reweighted model keeps the original current-week behavioral distance while adding on sensitivity to recent behavioral change.

Shortest paths were recomputed with $w_{ij}^{t,\mathrm{dyn}}$instead of the original edge weight $w_{ij}^{t}$. This preserves the original shortest-path formulation while incorporating temporal dependence. Abrupt behavioral deviations were captured through the week-to-week change term because sudden changes produce larger $\Delta z_{i}^{t}$values and increase edge cost when neighboring users do not show similar changes. Weekly and biweekly rows were compared for alignment with observed real-world transitions at different temporal granularities with Spearman correlation and cosine distance. Table 2.1 shows the results.

**Table 2.1** Results of temporal sensitivity analysis of GraphBeTraM using dynamic edge reweighting and temporal granularity comparison.

| **Model** | **Spearman ρ** | **P value** | **Cosine distance** |
| --- | --- | --- | --- |
| GraphBeTraM  (original model with weekly granularity) | 0.82 | <.001 | 0.05 |
| Temporal GraphBeTraM  (temporal edge-reweighted model with weekly granularity) | 0.74 | <.001 | 0.17 |
| Temporal GraphBeTraM  (temporal edge-reweighted model with bi-weekly granularity) | 0.67 | <.001 | 0.15 |
