# Supplementary material for: Graph-Based Modeling of Behavioral Transitions for Digital Health Applications: Trajectory Analysis Framework
Source: J Med Internet Res. 2026 Sep 4;28:e86629. doi: 10.2196/86629 (PMC13544619; doi:10.2196/86629)
Supplement: Supplementary file 3 [file jmir-v28-e86629-s003.docx]

**Multimedia Appendix 3 (Table).** Number of users transitioning from source state/ cluster in week 0 to target state/cluster in week 31. To model a 32-week behavioral transition period for 42 state/ cluster pairs: MVPA-focused active, Step-focused active, Predominantly sedentary, Highly active, Sedentary office active, Active and healthy eating, and Class-based active.

| **Source state or cluster** | **Target state or cluster** | **Frequency of users with actual transitions in the dataset** |
| --- | --- | --- |
| MVPA-focused active | Step-focused active | 280 |
| Step-focused active | MVPA-focused active | 376 |
| MVPA-focused active | Predominantly sedentary | 55 |
| Predominantly sedentary | MVPA-focused active | 139 |
| MVPA-focused active | Very active | 912 |
| Highly active | MVPA-focused active | 931 |
| MVPA-focused active | Sedentary office active | 769 |
| Sedentary office active | MVPA-focused active | 1211 |
| MVPA-focused active | Active and healthy eating | 290 |
| Active and healthy eating | MVPA-focused active | 212 |
| MVPA-focused active | Class-based active | 71 |
| Class-based active | MVPA-focused active | 56 |
| Step-Focused active | Sedentary | 126 |
| Sedentary | Step-focused active | 182 |
| Step-focused active | Highly active | 109 |
| Highly active | Step-focused active | 63 |
| Step-focused active | Sedentary office active | 796 |
| Sedentary office active | Step-focused active | 826 |
| Step-focused active | Active and healthy eating | 65 |
| Active and healthy eating | Step-focused active | 54 |
| Step-focused active | Class-based active | 24 |
| Class-based active | Step-focused active | 11 |
| Sedentary | Highly active | 62 |
| Highly active | Sedentary | 45 |
| Sedentary | Sedentary office active | 1528 |
| Sedentary office active | Sedentary | 1035 |
| Sedentary | Active and healthy eating | 86 |
| Active and healthy eating | Sedentary | 59 |
| Sedentary | Class-based active | 25 |
| Class-based active | Sedentary | 19 |
| Highly active | Sedentary office active | 848 |
| Sedentary office active | Highly active | 1055 |
| Highly active | Active and healthy eating | 443 |
| Active and healthy eating | Highly active | 326 |
| Highly active | Class-based active | 73 |
| Class-based active | Highly active | 59 |
| Sedentary during office hours | Active and healthy eating | 646 |
| Active and healthy eating | Sedentary office active | 540 |
| Sedentary office active | Class-based active | 170 |
| Class-based active | Sedentary office active | 148 |
| Active and healthy eating | Class-based active | 24 |
| Class-based active | Active and healthy eating | 23 |
