# Supplementary material for: Graph-Based Modeling of Behavioral Transitions for Digital Health Applications: Trajectory Analysis Framework
Source: J Med Internet Res. 2026 Sep 4;28:e86629. doi: 10.2196/86629 (PMC13544619; doi:10.2196/86629)
Supplement: Supplementary file 4 [file jmir-v28-e86629-s004.docx]

## **Multimedia Appendix 4 (Table).** Number of users at baseline (30 October 2023, N=36,574) and demographics of users in 7 behavioral states. MVPA: medium to vigorous physical activity.

|  | **MVPA-focused active** | **Step-focused active** | **Predominantly sedentary** | **Highly active** | **Sedentary office active** | **Active and healthy eating** | **Class-based active** |
| --- | --- | --- | --- | --- | --- | --- | --- |
| **n (%)** | 6433 (17.59) | 1841 (5.03) | 2569 (7.02) | 7145 (19.53) | 15,426 (42.17) | 2203 (6.02) | 957 (2.62) |
| **Age in years, mean (SD)** | 60.1 (11.5) | 48.2 (11.6) | 43.8 (10.6) | 59.4 (11.6) | 48.4 (11) | 55.3 (12.2) | 55.2 (11.2) |
| **Sex, n (%)** | | | | | | | |
| **Male** | 2632 (40.91) | 680 (36.94) | 910 (35.42) | 3256 (45.57) | 6123 (39.69) | 844 (38.31) | 82 (8.57) |
| **Female** | 3801 (59.09) | 1161 (63.06) | 1659 (64.58) | 3889 (54.43) | 9303 (60.31) | 1359 (61.69) | 875 (91.43) |
| **Ethnicity, n (%)** | | | | | | | |
| **Chinese** | 5548 (86.24) | 1512 (82.13) | 2111 (82.17) | 6266 (87.70) | 12825 (83.14) | 1972 (89.51) | 835 (87.25) |
| **Indian** | 213 (3.31) | 63 (3.42) | 86 (3.35) | 182 (2.55) | 622 (4.03) | 23 (1.04) | 15 (1.57) |
| **Malay** | 94 (1.46) | 38 (2.06) | 48 (1.87) | 60 (0.84) | 223  (1.45) | 33 (1.50) | 8 (0.84) |
| **Others** | 578 (8.99) | 228 (12.39) | 324 (12.61) | 637 (8.91) | 1756 (11.38) | 175 (7.95) | 99 (10.34) |
