# Supplementary material for: Graph-Based Modeling of Behavioral Transitions for Digital Health Applications: Trajectory Analysis Framework
Source: J Med Internet Res. 2026 Sep 4;28:e86629. doi: 10.2196/86629 (PMC13544619; doi:10.2196/86629)
Supplement: Supplementary file 5 [file jmir-v28-e86629-s005.docx]

## **Multimedia Appendix 5 (Table).** Behavioral Features in the dataset showing mean and median distributions across users (N=36,574) in the dataset. MVPA: medium to vigorous physical activity. VPA: vigorous physical activity.

| **Feature** | **Mean (SD)** | **Median (IQR)** |
| --- | --- | --- |
| Tracker usage | 6.1 (1.3) | 6.8 (5.8 – 7.0) |
| Ratio sedentary to steps | 0.2 (0.3) | 0.0 (-1.0 - 0.0) |
| Weekly MVPA | 1.3 (0.5) | 1.3 (0.9 – 1.7) |
| Ratio steps to MVPA | 0.1 (0.3) | 0.1 (-0.1 – 0.3) |
| Last contact | 0.4 (0.3) | 0.3 (0.3 – 0.6) |
| Sedentary routine | 310.6 (136.6) | 322.9 (210.4 – 413.0) |
| MVPA on weekends | 0.5 (0.3) | 0.5 (0.3 – 0.7) |
| Ratio weekday to weekend | -0.1 (0.5) | -0.1 (-0.4 – 0.2) |
| Healthy food and drink purchased | 0.1 (0.2) | 0.0 (0.0 – 0.0) |
| Types of exercise classes attended | 0.1 (0.5) | 0.0 (0.0 – 0.0) |
| Tracker syncing | 0.5 (0.2) | 0.4 (0.3 – 0.7) |
| Healthy groceries purchased | 0.7 (0.5) | 0.8 (0.0 – 1.1) |
| MVPA on weekdays | 0.5 (0.2) | 0.4 (0.3 – 0.6) |
| MVPA trend | -0.1 (0.4) | -0.0 (-0.2 – 0.1) |
| Steps tracked | 1.0 (0.1) | 1.0 (0.9 – 1.1) |
| Ratio VPA to MVPA | 0.1 (0.1) | 0.1 (0.0 – 0.2) |
