# Supplementary material for: Graph-Based Modeling of Behavioral Transitions for Digital Health Applications: Trajectory Analysis Framework
Source: J Med Internet Res. 2026 Sep 4;28:e86629. doi: 10.2196/86629 (PMC13544619; doi:10.2196/86629)
Supplement: Supplementary file 6 [file jmir-v28-e86629-s006.docx]

## **Multimedia Appendix 6 (Table).** Distribution of average feature values across users in 7 behavioral states (N=36,574). MVPA: medium to vigorous physical activity.

|  | **MVPA-focused active** | **Step-focused active** | **Predominantly sedentary** | **Highly active** | **Sedentary office active** | **Active and healthy eating** | **Class-based active** |
| --- | --- | --- | --- | --- | --- | --- | --- |
| **Tracker usage, mean (SD)** | 5.74 (1.5) | 3.42 (1.3) | 5.28 (1.6) | 6.76 (0.6) | 6.31 (1.0) | 6.55 (0.9) | 6.52 (0.9) |
| **Ratio sedentary to steps, mean (SD)** | 0.12 (0.3) | -0.27 (0.3) | -0.06 (0.3) | 0.61 (0.2) | 0.10 (0.3) | 0.31 (0.3) | 0.29 (0.3) |
| **Weekly MVPA, mean (SD)** | 1.66 (0.4) | 0.90 (0.4) | 0.58 (0.3) | 1.78 (0.4) | 1.03 (0.4) | 1.41 (0.5) | 1.44 (0.4) |
| **Ratio steps to MVPA, mean (SD)** | -0.26 (0.2) | -0.11 (0.2) | 0.33 (0.3) | 0.05 (0.1) | 0.26 (0.2) | 0.11 (0.3) | 0.10 (0.2) |
| **Last contact, mean (SD)** | 0.35 (0.2) | 0.98 (0.3) | 0.48 (0.3) | 0.26 (0.2) | 0.37 (0.3) | 0.31 (0.2) | 0.24 (0.2) |
| **Sedentary routine, mean (SD)** | 245.70 (123.9) | 350.90 (117.2) | 370.09 (106.1) | 181.89 (105.5) | 386.06 (98.5) | 286.12 (130.5) | 309.76 (128.6) |
| **MVPA on weekends** | 0.41 (0.2) | 0.59 (0.3) | 0.01 (0.06) | 0.44 (0.2) | 0.66 (0.2) | 0.50 (0.2) | 0.53 (0.2) |
| **Ratio weekday to weekend** | -0.11 (0.3) | -0.09 (0.6) | -0.98 (0.1) | -0.08 (0.3) | -0.005 (0.5) | -0.11 (0.4) | -0.09 (0.4) |
| **Healthy food and drink purchased, mean (SD)** | 0.02 (0.08) | 0.03 (0.1) | 0.03 (0.1) | 0.02 (0.07) | 0.03 (0.1) | 0.80 (0.3) | 0.07 (0.2) |
| **Types of exercise classes attended, mean (SD)** | 0.05 (0.2) | 0.02 (0.1) | 0.02 (0.2) | 0.04 (0.2) | 0.04 (0.2) | 0.05 (0.2) | 2.75 (1.2) |
| **Tracker syncing, mean (SD)** | 0.53 (0.2) | 0.25 (0.1) | 0.33 (0.2) | 0.63 (0.2) | 0.39 (1.0) | 0.54 (0.2) | 0.57 (0.2) |
| **Healthy groceries purchased, mean (SD)** | 0.54 (0.5) | 0.42 (0.4) | 0.74 (0.5) | 0.58 (0.6) | 0.78 (0.5) | 1.02 (0.5) | 0.68 (0.5) |
| **MVPA on weekdays, mean (SD)** | 0.35 (0.1) | 0.53 (0.2) | 0.64 (0.2) | 0.41 (0.2) | 0.54 (0.2) | 0.45 (0.2) | 0.50 (0.2) |
| **MVPA trend, mean (SD)** | 0.01 (0.3) | -0.53 (0.4) | 0.03 (0.6) | -0.02 (0.2) | -0.06 (0.4) | -0.04 (0.3) | -0.04 (0.3) |
| **Steps tracked, mean (SD)** | 0.89 (0.1) | 0.92 (0.1) | 0.95 (0.1) | 1.11 (0.1) | 1.01 (0.1) | 1.03 (0.1) | 1.04 (0.1) |
| **Ratio VPA to MVPA, mean (SD)** | 0.07 (0.07) | 0.08 (0.08) | 0.07 (0.09) | 0.13 (0.08) | 0.09 (0.08) | 0.09 (0.08) | 0.12 (0.1) |
