# Supplementary material for: Graph-Based Modeling of Behavioral Transitions for Digital Health Applications: Trajectory Analysis Framework
Source: J Med Internet Res. 2026 Sep 4;28:e86629. doi: 10.2196/86629 (PMC13544619; doi:10.2196/86629)
Supplement: Supplementary file 7 [file jmir-v28-e86629-s007.docx]

## **Multimedia Appendix 7 (Table).** Features and their frequency as high-variance features across 21 pairwise transitions formed from 7 behavioral states.

| **Features** | **High-variance frequency, n (%)** |
| --- | --- |
| Tracker usage | 15 (71.4) |
| Ratio sedentary to steps | 15 (71.4) |
| Weekly MVPA | 15 (71.4) |
| Ratio steps to MVPA | 10 (47.6) |
| Last contact | 9 (42.9) |
| Sedentary routine | 9 (42.9) |
| MVPA on weekends | 9 (42.9) |
| Ratio weekday to weekend | 6 (28.6) |
| Healthy food and drink purchased | 6 (28.6) |
| Types of exercise classes attended | 6 (28.6) |
| Tracker synced | 5 (23.8) |
| Healthy groceries purchased | 4 (19) |
| MVPA on weekdays | 2 (9.5) |
| MVPA trend | 2 (9.5) |
| Steps tracked | 1 (4.8) |
| Ratio VPA to MVPA | 0 (0) |
