# Supplementary material for: Graph-Based Modeling of Behavioral Transitions for Digital Health Applications: Trajectory Analysis Framework
Source: J Med Internet Res. 2026 Sep 4;28:e86629. doi: 10.2196/86629 (PMC13544619; doi:10.2196/86629)
Supplement: Supplementary file 8 [file jmir-v28-e86629-s008.docx]

## **Multimedia Appendix 8.** Validation of Graph Model Construction and Structure.

**Table 8.1.** Sensitivity Analysis of Graph-Construction Parameters.

The Euclidean distance provides an interpretable basis for nearest-neighbor graph construction in a PCA-reduced feature space, where observations are represented as principal component score vectors and the Euclidean distance provides an interpretable basis for nearest-neighbor graph construction. Metrics were computed across k=5-20 for all 32 weekly graphs. Modularity metric reflects stability of community structure, while clustering coefficient reflects stability of local connectivity patterns.

| **Metric** | **Observed range** | **Interpretation** |
| --- | --- | --- |
| Modularity | 0.666–0.703 | Stable community structure across k=5-20 for all 32 weekly graphs |
| Clustering coefficient | 0.256–0.258 | Stable local neighborhood cohesion across k=5-20 for all 32 weekly graphs |

**Table 8.2.** Stability Analysis of Graph Structure and Temporal Coherence.

Temporal graph-structure stability analysis across 32 weekly graphs. Modularity metric reflects stability of community structure, while clustering coefficient reflects stability of local connectivity patterns.

| **Metric** | **Mean (SD)** | **Median (IQR)** |
| --- | --- | --- |
| Modularity | 0.660 (0.019) | 0.663 (0.647–0.674) |
| Clustering coefficient | 0.269 (0.004) | 0.269 (0.265–0.272) |
