# Supplementary material for: Graph-Based Modeling of Behavioral Transitions for Digital Health Applications: Trajectory Analysis Framework
Source: J Med Internet Res. 2026 Sep 4;28:e86629. doi: 10.2196/86629 (PMC13544619; doi:10.2196/86629)
Supplement: Supplementary file 9 [file jmir-v28-e86629-s009.docx]

## **Multimedia Appendix 9 (Table).** Sensitivity Analysis of Missing Data in Shortest Paths.

Shortest-path node-hiding sensitivity analysis. N= 36,574 users.

| **% hidden intermediate nodes** | **Proportion of users (n, %)** | **Spearman ρ** | **P value** | **Cosine distance** |
| --- | --- | --- | --- | --- |
| 10% | 34,661 (95) | 0.78 | <.001 | 0.05 |
| 20% | 6256 (17.1) | 0.65 | <.001 | 0.07 |
| 40% | 1314 (3.6) | 0.63 | <.001 | 0.07 |
