# Supplementary material for: Graph-Based Modeling of Behavioral Transitions for Digital Health Applications: Trajectory Analysis Framework
Source: J Med Internet Res. 2026 Sep 4;28:e86629. doi: 10.2196/86629 (PMC13544619; doi:10.2196/86629)
Supplement: Supplementary file 10 [file jmir-v28-e86629-s010.docx]

## **Multimedia Appendix 10 (Table).** Transition-matrix benchmarking against non-graph baselines.

Transition-matrix alignment between observed and model-predicted behavioral transitions.

| **Method** | **Spearman ρ** | **P value** | **Cosine distance** |
| --- | --- | --- | --- |
| GraphBeTraM | 0.82 | <.001 | 0.05 |
| PCA-distance nearest-centroid baseline | 0.65 | <.001 | 0.06 |
| Empirical Markov baseline | 0.61 | <.001 | 0.05 |
