# Supplementary material for: Graph-Based Modeling of Behavioral Transitions for Digital Health Applications: Trajectory Analysis Framework
Source: J Med Internet Res. 2026 Sep 4;28:e86629. doi: 10.2196/86629 (PMC13544619; doi:10.2196/86629)
Supplement: Supplementary file 11 [file jmir-v28-e86629-s011.docx]

## **Multimedia Appendix 11.** Stratified analysis by demographic characteristics.

**Table 11.1.** Demographic stratified analysis showing Fisher’s z transformation mean Spearman correlation and mean cosine distance across n=23 transition-subgroup comparisons.

| **Feature** | **Proportion of stable transition-subgroup comparisons, n (%)** | **Spearman ρ,**  **mean (SD)**  **(n=23 transition-subgroup)** | **Cosine distance,**  **mean (SD)**  **(n=23 transitions)** | **Stability*** |
| --- | --- | --- | --- | --- |
| Weekly MVPA | 21 (91) | 0.98 (2.36) | 0.23 (0.27) | Generally stable |
| Healthy food and drink purchased | 14 (61) | 0.93 (1.69) | 0.41 (0.39) | Partially stable |
| Ratio sedentary to steps | 12 (52) | 0.96 (2.54) | 0.36 (0.29) | Partially stable |
| Last contact | 12 (52) | 0.57 (0.60) | 0.42 (0.15) | Partially stable |
| Tracker usage | 10 (43) | 0.68 (0.46) | 0.40 (0.18) | Partially stable |
| Ratio steps to MVPA | 9 (39) | 0.57 (0.66) | 0.49 (0.42) | Variable |
| Sedentary routine | 8 (35) | 0.84 (2.05) | 0.51 (0.39) | Variable |
| MVPA on weekends | 1 (4) | 0.32 (0.47) | 0.69 (0.29) | Highly variable |
| Ratio weekday to weekend | 1 (4) | 0.26 (0.51) | 0.83 (0.34) | Highly variable |
| Types of exercise classes attended | 1 (4) | 0.44 (0.48) | 0.87 (0.39) | Highly variable |

^*^Transition-subgroup comparison is stable if Spearman ρ ≥ 0.70 and cosine distance≤0.50. Feature is Generally stable if stable in ≥70% comparisons; Partially stable if stable in ≥40%; Variable if stable in ≥ 10%; and Highly variable if not.

**Table 11.2.** Overview of transition-subgroup combinations included in stratified analysis to assess feature-level stability across combinations. A checkmark indicates that the feature met both predefined stability criteria for the corresponding transition-subgroup comparison (Spearman ρ ≥ 0.70 and cosine distance ≤ 0.50). Feature columns are ordered from highest to lowest stability across the 23 valid transition- subgroup comparisons.

| Source state or cluster | Target state or cluster | Subgroup | User count* (n) | Feature meets stability criteria (Spearman ρ ≥ 0.70 and cosine distance ≤ 0.50) across subgroup transition comparisons with at least 30 users having these transitions | | | | | | | | | |
| --- | --- | --- | --- | --- | --- | --- | --- | --- | --- | --- | --- | --- | --- |
|  |  |  |  | Weekly MVPA | Healthy food and drink purchased | Ratio sedentary to steps | Last contact | Tracker usage | Ratio steps to MVPA | Sedentary routine | MVPA on weekends | Ratio weekday to weekend | Types of exercise classes attended |
| Predominantly sedentary | Highly active | Age 40–59 | 34 | ✓ |  | ✓ | ✓ |  |  | ✓ |  |  |  |
|  |  | Non-Chinese | 62 | ✓ |  | ✓ | ✓ | ✓ |  |  |  |  |  |
|  |  | Female | 39 | ✓ |  | ✓ | ✓ | ✓ |  |  |  |  |  |
|  | Active and healthy eating | Age 40–59 | 52 | ✓ | ✓ | ✓ | ✓ | ✓ | ✓ |  |  |  |  |
|  |  | Non-Chinese | 86 | ✓ | ✓ | ✓ | ✓ | ✓ | ✓ | ✓ |  |  | ✓ |
|  |  | Female | 57 | ✓ | ✓ | ✓ | ✓ | ✓ |  |  |  |  |  |
| Highly active | Active and healthy eating | Age 40–59 | 113 | ✓ | ✓ |  |  |  |  |  |  |  |  |
|  |  | Age ≥60 | 317 | ✓ | ✓ |  |  |  |  |  |  |  |  |
|  |  | Non-Chinese | 443 | ✓ | ✓ |  |  |  |  |  |  |  |  |
|  |  | Female | 268 | ✓ | ✓ |  |  |  |  |  |  |  |  |
|  |  | Male | 175 |  | ✓ |  |  |  |  |  |  |  |  |
| Sedentary office active | Highly active | Age 21–39 | 102 | ✓ |  | ✓ | ✓ |  | ✓ | ✓ |  |  |  |
|  |  | Age 40–59 | 544 | ✓ |  | ✓ | ✓ |  | ✓ | ✓ |  |  |  |
|  |  | Age ≥60 | 409 | ✓ |  | ✓ |  |  | ✓ | ✓ |  |  |  |
|  |  | Non-Chinese | 1055 | ✓ |  | ✓ |  |  | ✓ | ✓ |  |  |  |
|  |  | Female | 583 | ✓ |  | ✓ |  |  | ✓ | ✓ |  |  |  |
|  |  | Male | 472 | ✓ |  | ✓ |  |  | ✓ | ✓ |  |  |  |
|  | Active and healthy eating | Age 21–39 | 70 |  | ✓ |  |  | ✓ |  |  |  |  |  |
|  |  | Age 40–59 | 377 | ✓ | ✓ |  |  | ✓ | ✓ |  |  |  |  |
|  |  | Age ≥60 | 199 | ✓ | ✓ |  | ✓ |  |  |  |  |  |  |
|  |  | Non-Chinese | 646 | ✓ | ✓ |  | ✓ | ✓ |  |  |  |  |  |
|  |  | Female | 420 | ✓ | ✓ |  | ✓ | ✓ |  |  |  | ✓ |  |
|  |  | Male | 226 | ✓ | ✓ |  | ✓ | ✓ |  |  | ✓ |  |  |

* Other transition- subgroup combinations had n < 30 users and thus were not valid for analysis.
