# Supplementary material for: Graph-Based Modeling of Behavioral Transitions for Digital Health Applications: Trajectory Analysis Framework
Source: J Med Internet Res. 2026 Sep 4;28:e86629. doi: 10.2196/86629 (PMC13544619; doi:10.2196/86629)
Supplement: Supplementary file 12 [file jmir-v28-e86629-s012.docx]

## **Multimedia Appendix 12.** **Transition Scenario 2 - Sedentary Office Active to Highly Active**

Figure 12.1 shows GraphBeTraM’s output for sedentary office active state to highly active state. In summary table, tracker usage has the earliest onset (step 2: early). In contrast, sedentary routine and ratio sedentary to steps appear more resistant to change (step 5: late). Weekly MVPA shows a very high magnitude of change (very high:1.61), while most display moderate to fast rate of change (0.14 - 0.20).

**Figure 12.1.** Outcome of GraphBeTraM for transition from sedentary office active state to highly active state, showing: heatmap of high-variance features; category thresholds for onset, magnitude and rate of change; observation summary of onset, magnitude, and rate of change. MVPA: medium to vigorous physical activity; S: source node; T: target node.

In Table 12.1, most features are validated except tracker usage (ρ=0.53; *P*=.14, cosine distance=0.54) which reflects context-dependency, suggesting that the relevance of tracker usage varies across different user groups or transition types. Sedentary routine reflects patterns specific to the sedentary office active state, potentially driven by time-constrained routines such as work-related commitments. Syncing the tracker (tracker synced – step 4 : mid; medium:1.14; moderate:0.14) more often could be encouraged, together with increasing MVPA instead of steps (weekly MVPA step 4: mid; medium:1.19; moderate: 0.15), though both might be slower changes to achieve. Later, more steps (ratio sedentary to steps step 5: late; medium:1.55; fast:0.19) and decreasing sedentary time (sedentary routine step 5: late; medium:1.33; fast:0.17), could be encouraged as users in the sedentary office active state might find it harder to break sedentary periods due to fixed working times.

**Table 12.1.** Validation of the feature transition pathways showing Spearman correlation and cosine distance scores for sedentary office active state to highly active state (n=1055).

| **Features** | **Personalized shortest path analysis** | | | **Real-World pathways** | | |
| --- | --- | --- | --- | --- | --- | --- |
|  | **Spearman ρ** | ***P* value** | **Cosine distance** | **Spearman ρ** | ***P* value** | **Cosine distance** |
| Tracker usage | 0.93 | <.01 | 0.16 | 0.53 | .14 | 0.54 |
| Tracker synced | 0.96 | <.001 | 0.11 | 0.88 | <.01 | 0.09 |
| Ratio sedentary to steps | 1.00 | <.001 | 0.07 | 1.00 | <.001 | 0.04 |
| Weekly MVPA | 1.00 | <.001 | 0.05 | 1.00 | <.001 | 0.04 |
| Sedentary routine | 1.00 | <.001 | 0.10 | 1.00 | <.001 | 0.03 |
| Ratio steps to MVPA | 0.96 | <.001 | 0.09 | 0.92 | <.001 | 0.06 |
