# Supplementary material for: Graph-Based Modeling of Behavioral Transitions for Digital Health Applications: Trajectory Analysis Framework
Source: J Med Internet Res. 2026 Sep 4;28:e86629. doi: 10.2196/86629 (PMC13544619; doi:10.2196/86629)
Supplement: Supplementary file 13 [file jmir-v28-e86629-s013.docx]

## **Multimedia Appendix 13. Transition Scenario 3 – Predominantly Sedentary to Highly Active**

Figure 13.1 presents GraphBeTraM’s output for predominantly sedentary state to highly active state. In summary table, tracker usage shows the earliest onset of change (step 2: early), potentially indicating more responsiveness to change. Given the strong contrast between the 2 states in both physical activity and engagement levels, we observe a high magnitude but fast rate of change across key features such as weekly MVPA, ratio sedentary to steps, and patterns related to active days like ratio weekday to weekend. In contrast, a time-specific feature like sedentary routine shows the lowest rate of change (Moderate: 0.15), likely due to the absence of time-dependent characteristics in these states.

**Figure 13.1.** Outcome of GraphBeTraM for transition between predominantly sedentary state to highly active state (n=62) showing: heatmap of high-variance features; category thresholds for onset, magnitude and rate of change; observation summary of onset, magnitude, and rate of change. MVPA: medium to vigorous physical activity; S: source node; T: target node.

In Table 13.1, most features were validated by both approaches except ratio weekday to weekend (ρ=0.50; *P*=.17, cosine distance=0.85), MVPA on weekends (ρ=-0.27; *P*=.49, cosine distance=0.95), and sedentary routine (ρ=-0.69; *P*=.05, cosine distance=0.46). These features reflect variations in users’ physical activity patterns depending on the time of day. Their partial validation indicates their relevance may depend on the user group or transition context. Although they may capture behavioral changes in some individuals, they do not consistently distinguish users undergoing this transition. It might be recommended to implement initiatives to encourage users to increase Tracker Usage as it shows an early onset (step2: early) and could be quick to achieve (fast: 0.18). Sedentary routine, with a mid-onset (step4: mid), perhaps could be achieved by suggesting easy movement routines to break sedentary patterns. Later, the user could be encouraged to progress to increase steps and MVPA.

**Table 13.1.** Validation of the feature transition pathways showing Spearman correlation and cosine distance scores for predominantly sedentary state to highly active state (n=62).

| **Features** | **Personalized shortest path analysis** | | | **Real-World pathways** | | |
| --- | --- | --- | --- | --- | --- | --- |
|  | **Spearman ρ** | ***P* value** | **Cosine distance** | **Spearman ρ** | ***P* value** | **Cosine distance** |
| Tracker usage | 0.89 | <.01 | 0.20 | 0.80 | <.001 | 0.42 |
| MVPA on weekends | -1.00 | <.001 | 1.37 | -0.27 | .49 | 0.95 |
| Ratio weekday to weekend | 1.00 | <.001 | 0.43 | 0.50 | .17 | 0.85 |
| Ratio sedentary to steps | 1.00 | <.001 | 0.00 | 0.88 | <.01 | 0.28 |
| Weekly MVPA | 1.00 | <.001 | 0.01 | 0.93 | <.001 | 0.10 |
| Sedentary routine | 0.89 | <.01 | 0.04 | -0.69 | .05 | 0.46 |
