# Supplementary material for: Graph-Based Modeling of Behavioral Transitions for Digital Health Applications: Trajectory Analysis Framework
Source: J Med Internet Res. 2026 Sep 4;28:e86629. doi: 10.2196/86629 (PMC13544619; doi:10.2196/86629)
Supplement: Supplementary file 14 [file jmir-v28-e86629-s014.docx]

## **Multimedia Appendix 14. Transition Scenario 4 - Predominantly Sedentary to Active and Healthy Eating**

In Figure 14.1, we present the output of GraphBeTraM applied to the transition pathway between centroid users from predominantly sedentary state to active and healthy eating state focusing on high-variance features. In summary table, tracker usage shows the earliest behavioral shift (onset step 2: early). Magnitude of change is high for all features and rate of change of healthy food and drink purchased and ratio sedentary to steps is slightly lower (moderate:0.17) than others (fast:0.19 – 0.20).

**Figure 14.1.** Outcome of GraphBeTraM for transition from predominantly sedentary state to active and healthy eating state (n=86) showing: heatmap of high-variance features; category thresholds for onset, magnitude and rate of change; observation summary of onset, magnitude, and rate of change. MVPA: medium to vigorous physical activity; S: source node; T: target node.

In Table 14.1, most features were validated except MVPA on weekends (ρ=-0.96, *P*<.001) and tracker usage (ρ=0.00, *P*>.99). These features capture nuances in users’ physical activity patterns, suggesting context dependency. While they may reflect behavioral change for some, they do not consistently distinguish users along this specific transition. We observe that the states have quite differing behavior profiles, with changes ranging from high:1.49 (weekly MVPA) to 1.22 (healthy food and drink purchased). It might be advisable to start with weekly MVPA (step4: mid) and ratio sedentary to steps (step4: mid), though weekly MVPA (fast: 0.21) might be faster to achieve than ratio sedentary to steps (moderate:0.17). Increasing tracker usage (tracker usage: step 2: early; high: 1.31; fast: 0.19) could also be recommended. Healthy food and drink purchased could be met with a high resistance to change (step5: late; high: 1.22; moderate: 0.17), thus starting to encourage it earlier might be recommended.

**Table 14.1.** Validation of the feature transition pathways showing Spearman correlation and cosine distance scores for predominantly sedentary state to active and healthy eating state (n=86)

| **Features** | **Personalized shortest path analysis** | | | **Real-World pathways** | | |
| --- | --- | --- | --- | --- | --- | --- |
|  | **Spearman ρ** | ***P* value** | **Cosine distance** | **Spearman ρ** | ***P* value** | **Cosine distance** |
| MVPA on weekends | -0.96 | <.001 | 1.13 | 0.90 | <.001 | 0.50 |
| Ratio weekday to weekend | 1.00 | <.001 | 0.07 | 0.76 | .03 | 0.67 |
| Healthy food and drink purchased | 0.75 | .05 | 0.11 | 0.98 | <.001 | 0.11 |
| Tracker usage | 0.00 | >.99 | 0.68 | 0.95 | <.001 | 0.03 |
| Ratio sedentary to steps | 1.00 | <.001 | 0.01 | 0.71 | .05 | 0.18 |
| Weekly MVPA | 0.89 | <.01 | 0.05 | 0.95 | <.001 | 0.05 |
