# Supplementary material for: Graph-Based Modeling of Behavioral Transitions for Digital Health Applications: Trajectory Analysis Framework
Source: J Med Internet Res. 2026 Sep 4;28:e86629. doi: 10.2196/86629 (PMC13544619; doi:10.2196/86629)
Supplement: Supplementary file 15 [file jmir-v28-e86629-s015.docx]

## **Multimedia Appendix 15. Transition Scenario 5 - Highly Active to Active and Healthy Eating**

Figure 15.1 presents the model’s output along the transition pathway between centroid users, from the highly active state to the active and healthy eating state, focusing on shifts in high-variance behavioral features. In summary table, as both states represent active behaviors, we observe specific to this transition a low magnitude of change and slow rate of change for weekly MVPA (onset of change – step5: very late; magnitude of change – low:0.55; rate of change – slow:0.09). sedentary behavior and healthy food and drink purchased display late onset of change (step 4: late and step 5: very late respectively) which might be due to resistance to change in sedentary behavior as well as being a very characteristic feature to the target cluster, respectively.

**Figure 15.1** Outcome of GraphBeTraM for transition from highly active state to active and healthy eating state (n=443) showing: heatmap of high-variance features; category thresholds for onset, magnitude and rate of change; observation summary of onset, magnitude, and rate of change. MVPA: medium to vigorous physical activity; S: source node; T: target node.

In Table 15.1, weekly MVPA and healthy food and drink purchased were identified as key features aligned with the observed transitions. This suggests that these patterns are consistent across users, making them reliable indicators of behavioral change for this transition. We observe that the magnitude of change (ranged from low: 0.48 (tracker synced) to high:1.3 (healthy food and drink purchased)) and rate of change (ranged from slow:0.08 (tracker synced) to fast:0.22 (healthy food/drink purchased)) are quite diverse in this transition. There are no quick wins here but perhaps a persistent nudging strategy to increase healthy food or drink purchases could be suggested.

**Table 15.1.** Validation of the feature transition pathway showing Spearman correlation and cosine distance scores for highly active state to active and healthy eating state (n=443).

| **Features** | **Personalized shortest path analysis** | | | **Real-World pathways** | | |
| --- | --- | --- | --- | --- | --- | --- |
|  | **Spearman ρ** | ***P* value** | **Cosine distance** | **Spearman ρ** | ***P* value** | **Cosine distance** |
| Sedentary routine | 0.89 | <.01 | 0.09 | -0.21 | .64 | 0.90 |
| Healthy food and drink purchased | 0.79 | .03 | 0.08 | 0.99 | <.001 | 0.16 |
| Ratio sedentary to steps | 1.00 | <.001 | 0.04 | 0.50 | .25 | 0.46 |
| Weekly MVPA | 0.96 | <.001 | 0.10 | 0.71 | .07 | 0.36 |
| Tracker synced | 0.68 | .09 | 0.30 | 0.36 | .43 | 0.58 |
